# A gene expression assay for simultaneous measurement of microsatellite instability and anti-tumor immune activity

**DOI:** 10.1186/s40425-018-0472-1

**Published:** 2019-01-21

**Authors:** Patrick Danaher, Sarah Warren, SuFey Ong, Nathan Elliott, Alessandra Cesano, Sean Ferree

**Affiliations:** NanoString Technologies®, Inc, 530 Fairview Ave. N, Seattle, Washington, 98109 USA

**Keywords:** MMRd, MSI, Biomarker, Diagnostic, Checkpoint inhibitors, TIS

## Abstract

**Background:**

Clinical benefit from checkpoint inhibitors has been associated in a tumor-agnostic manner with two main tumor traits. The first is tumor antigenicity, which is typically measured by tumor mutation burden, microsatellite instability (MSI), or Mismatch Repair Deficiency using gene sequence platforms and/or immunohistochemistry. The second is the presence of a pre-existing adaptive immune response, typically measured by immunohistochemistry (e.g. single analyte PD-L1 expression) and/or gene expression signatures (e.g. tumor “inflamed” phenotype). These two traits have been shown to provide independent predictive information. Here we investigated the potential of using gene expression to predict tumor MSI, thus enabling the measurement of both tumor antigenicity and the level of tumor inflammation in a single assay, possibly reducing sample requirement, turn-around time, and overall cost.

**Methods:**

Using The Cancer Genome Atlas RNA-seq datasets with the greatest MSI-H incidence, i.e. those from colon (*n* = 208), stomach (*n* = 269), and endometrial (*n* = 241) cancers, we trained an algorithm to predict tumor MSI from under-expression of the mismatch repair genes MLH1, PMS2, MSH2, and MSH6 and from 10 additional genes with strong pan-cancer associations with tumor hypermutation. The algorithms were validated on the NanoString nCounter™ platform in independent cohorts of colorectal (*n* = 52), endometrial (*n* = 11), and neuroendocrine (*n* = 4) tumors pre-characterized using the MMR immunohistochemistry assay.

**Results:**

In the validation cohorts, the algorithm showed high prediction accuracy of tumor MSI status, with sensitivity of at least 88% attained at thresholds chosen to achieve 100% specificity. Furthermore, MSI status was compared to the Tumor Inflammation Signature (TIS), an analytically validated diagnostic assay which measures a suppressed adaptive immune response in the tumor and enriches for response to immune checkpoint blockade. TIS score was largely independent of MSI status, suggesting that measuring both parameters may identify more patients that would respond to immune checkpoint blockade than either assay alone.

**Conclusions:**

Development of a gene expression signature of MSI status raises the possibility of a combined diagnostic assay on a single platform which measures both tumor antigenicity and presence of a suppressed adaptive immune response. Such an assay would have significant advantages over multi-platform assays for both ease of use and turnaround time and could lead to a diagnostic test with improved clinical performance.

**Electronic supplementary material:**

The online version of this article (10.1186/s40425-018-0472-1) contains supplementary material, which is available to authorized users.

## Background

The clinical benefit of checkpoint inhibitors varies widely between patients and only a small subset experience durable disease remission upon treatment. Response to checkpoint inhibition has been shown to associate with two main biological axes: tumor antigenicity, typically measured by tumor mutation burden [1] or microsatellite instability (MSI) [2, 3] using sequencing platforms or qPCR, and the presence of an adaptive anti-tumor immune response, typically measured by gene expression signatures of tumor inflammation [4, 5] or by immunohistochemistry [6]. Because tumor antigenicity and the magnitude of the adaptive immune response in the tumor microenvironment are only weakly correlated [7], more accurate prediction of immunotherapy response should be possible by measuring and integrating both variables together. However, in a clinical setting, performing multiple assays using different platforms is often impractical due to increased tissue requirement, turn-around time, and cost. Here we investigate the ability of gene expression to predict tumor MSI, enabling measurement of tumor antigenicity and tumor inflammation in a single assay.

DNA mismatch repair deficiency (MMRd) has been observed in most cancer types in The Cancer Genome Atlas (TCGA), and occurs in more than 5% of adrenal, rectal, colon, stomach, and endometrial tumors [8]. Tumors with this phenotype develop both point and frameshift mutations at an increased rate and are often described as “hypermutated”. The failure of mismatch repair (MMR) to correct replication errors at short repeated DNA sequences can lead to the phenomenon of high-level MSI (MSI-H). MSI-H cancers have distinct clinical behavior, which has led to widespread MSI testing in cancers where MSI-H is common. In colorectal cancer, the MSI-H phenotype demonstrates association with proximal tumor localization, a dense local lymphocyte infiltration, and a low frequency of distant organ metastasis [9]. Moreover, MSI-H colorectal cancers have a better prognosis than their microsatellite-stable (MSS) counterparts [10]. Despite this, diminished responsiveness of MSI-H colorectal cancer patients towards chemotherapy has been shown in several studies, perhaps as a result of the elevated mutation rate more frequently giving rise to chemotherapy resistant clones [11]. In the era of immunotherapy, MMRd has gained greater relevance as a cause of hypermutation potentiating anti-tumor immune responses which may be enhanced by checkpoint inhibition [3]. Importantly, the frame-shift mutations that accrue in MMRd tumors can cause greater immunogenicity by leading to a shift in the protein coding sequence of the entire transcript downstream of the mutation site, whereas point mutations only create a potential neoantigen at the site of the mutation [12]. Thus, it is hypothesized that the high pan-cancer clinical efficacy of checkpoint inhibitors in MMRd tumors may arise more from their high rate of frameshift mutations than from their total tumor mutation burden.

MMRd often arises from loss of protein expression of at least 1 of 4 genes essential for MMR: MLH1, MSH2, MSH6, and PMS2. Lost expression of these proteins can arise either from acquired somatic mutations [13] or from germline mutations associated with Lynch syndrome [14]. In tumors with intact sequences for these genes, loss of protein expression can follow loss of mRNA expression. A common cause of lost mRNA expression in these genes is the CpG island methylator phenotype (CIMP), which is associated with widespread methylation across the genome and frequently silences DNA repair genes [15–20]. Loss of MMR activity due to microRNA-induced downregulation of MSH2 has also been observed in colorectal tumors [21]. MMRd can be detected by measuring either its cause or its effect. Immunohistochemistry (IHC) is used to measure loss of expression of proteins essential to the MMR machinery, and PCR and sequencing are used to measure MSI [22], one embodiment of genomic “scarring” which occurs as a consequence of MMRd.

The biology underlying MMRd provides two opportunities for capturing MMRd with gene expression data. First, loss of expression of MMR genes may be used to detect cases of MMRd resulting from transcriptional dysregulation. Second, if it is assumed that MMRd and CIMP exert broad and consistent influence on the transcriptome, then a data-driven predictor of hypermutation based on RNA expression patterns may also be possible. Here we employed both of these approaches in TCGA colon, endometrial, and stomach datasets to derive two independent predictors of tumor MSI, which were then combined in a single optimized predictor. We then evaluated these predictors in independent datasets collected using the NanoString nCounter gene expression platform (NanoString Technologies, Inc., Seattle, Washington, USA). The present study demonstrates the possibility of measuring both mechanisms of checkpoint inhibitor sensitivity, anti-tumor immunity and tumor antigenicity, simultaneously using a single gene expression assay.

## Methods

### Processing of TCGA datasets

The TCGA colon adenocarcinoma (COAD), stomach adenocarcinoma (STAD) and uterine carcinoma (UCEC) datasets were selected for analysis based on the > 15% incidence of MSI-H in these tumor types, making them the most relevant and best statistically powered TCGA datasets for our analyses.

TCGA datasets were downloaded from the Broad Firehose webpage [23]. RNASeq data were downloaded in RSEM-normalized format and log2-transformed. Each tumor’s mutation burden was calculated as the number of non-synonymous mutations in its whole exome DNA sequencing data; MSI status was taken verbatim from the TCGA source data, which used the MSI-Mono-Dinucleotide Assay qPCR panel to measure the length of the microsatellite regions.

### MMRd assay in commercial colorectal carcinoma samples

MSI-H and MSS colorectal cancer tumor samples in formalin-fixed paraffin-embedded (FFPE) blocks were purchased from iSpecimen (Lexington, Massachusetts, USA). MMR status was determined by the original clinical source using IHC for MLH1, MSH2, MSH6, and PMS2. MMR status was confirmed in samples where the original pathological assessment was discordant with the gene expression results by independent IHC staining and pathological review by PhenoPath Laboratories, PLLC (Seattle, Washington, USA).

### MMRd assay in commercial endometrial cancer samples

MMR status in the commercial endometrial cancer samples (also purchased from iSpecimen) was determined by IHC performed at PhenoPath Laboratories, PLLC (Seattle, Washington, USA). Antibody clones used were MSH2 (mouse monoclonal FE11, catalog # M3639; Dako), MSH6 (rabbit monoclonal EP49, catalog # M3646; Dako), MLH1 (mouse monoclonal ES05, catalog # M3640; Dako) and PMS2 (rabbit monoclonal EP51, catalog # M3647; Dako) (Agilent Technologies, Inc., Santa Clara, California, USA). All samples were stained with hematoxylin and eosin to allow for morphological evaluation. MMR status was reviewed by a board-certified pathologist and reported as “no loss of expression” or “loss of expression.”

### NanoString assay and normalization

Samples were run using the standard nCounter Gene Expression assay methodology [24] (NanoString Technologies, Inc., Seattle, Washington, USA). Total RNA was extracted from each FFPE tumor sample using the Qiagen FFPE RNeasy kit (Qiagen, Inc., Hilden, Germany). A total of 100 ng of RNA was hybridized with the PanCancer IO 360™ gene expression panel (NanoString Technologies, Inc., Seattle, Washington, USA), which contained the genes used in both the MSI Predictor algorithm and the Tumor Inflammation Signature. Downstream processing and data collection followed the manufacturer’s instructions.

Both NanoString datasets were normalized such that the mean log2 expression of 10 housekeeping genes was constant across all samples. All analyses used log2-transformed data.

### Calculation of MSI algorithms in NanoString data

Platform differences prevented directly applying the TCGA-trained algorithms to NanoString data. Because gene expression platforms differ in the efficiency with which they measure each target sequence, platform effects can be well-modelled by a constant shift in each gene’s log-scale normalized expression. Therefore, to apply the algorithms described in the Results section to NanoString data, the magnitudes of these platform effects were estimated for each MMR gene and for the Hypermutation Predictor score. To preserve the integrity of this dataset as an unbiased test set for the algorithms, all the calibration parameters were estimated using unsupervised methods without reference to the known MSI calls. The R library Mclust [25] was used to fit a two-component Gaussian mixture model to each MMR gene’s log2-transformed, normalized expression and to the Hypermutation Predictor score. For the MMR genes, the mean of the higher of the two clusters was the estimate of the mean expression level in non-hypermutated samples; for the Hypermutation Predictor score, it was the mean of the lower of the two clusters. Apart from these mean estimates, all other parameters needed to produce algorithm scores were calculated from TCGA data without reference to the validation dataset.

### Sequencing

Whole exome sequencing was performed on a subset of discordant samples via Agilent SureSelect Exome library preparation with Illumina HiSeq. SNP analysis was performed using the dbSNP database (NCI).

### Intended use and reproducible research

The MSI algorithms are intended for research use only and are not for use in diagnostic procedures. Data and R code for generating all figures in this paper can be found in Additional files 1 and 2.

## Results

### Loss of MMR gene expression predicts tumor MSI and hypermutation status

**Fig. 1:**
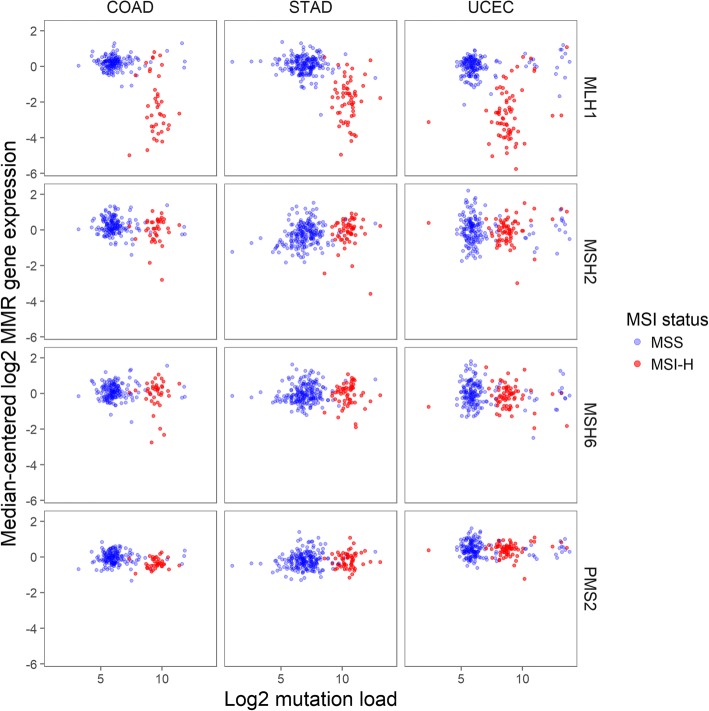
Expression of tumor mismatch repair genes versus tumor mutation burden in each TCGA dataset. Each column shows data from a single cancer type, and each row shows data from a single gene. Color denotes tumor microsatellite instability (MSI) status as reported in the TCGA database

Because loss of protein expression for any of the MMR genes MLH1, MSH2, MSH6, or PMS2 is sufficient to identify the majority of tumors with MSI-H status, we hypothesized that loss of mRNA expression for any of these genes would provide a surrogate measurement of tumor MSI status. Plotting MMR gene expression against mutation burden and MSI status revealed the strong association between these 3 phenomena (Fig. 1). Loss of MMR gene expression was strongly predictive of both MSI-H and hypermutation status and almost never occurred in cancers without MSI-H and hypermutation status.

In all 3 tumor types investigated (colon, stomach, and endometrial), a cluster of hypermutated tumors was visibly distinct from a primary cluster of samples with a lower mutation rate (Fig. 1). In each dataset, these hypermutated tumors were strongly enriched for MSI-H. Each dataset also contained a small third cluster of tumors with at least double the mutation burden of hypermutated tumors. Interestingly, while these “ultramutated” tumors in the endometrial cohort were often MSS, TCGA sequencing data confirmed each of these tumors to have mutations in one of the polymerase genes POLE or POLD1, consistent with a mechanism in which defective polymerase leads to widespread errors in DNA replication [26, 27]. Importantly, the average mutation burden within a given cluster is not preserved across tumor types; for example, non-hypermutated (typical) stomach cancers have 2 times the mutation rate of non-hypermutated endometrial cancers.

Loss of expression of the 4 MMR genes is also apparent within each cancer type (Fig. 1). MLH1 was by far the most frequently under-expressed of these genes. In TCGA database, MLH1 expression loss occurred in 16% of colon cancers, 20% of stomach cancers, and 29% of endometrial cancers. MLH1 loss on its own was a sensitive biomarker, detecting two thirds or more of the hypermutation cases in each of these cancer types. Expression loss in the other 3 MMR genes detected a small number of additional hypermutated/MSI-H samples not captured by MLH1 expression loss: MSH2 expression loss detected 5 additional MSI-H tumors in these 4 datasets, MSH6 expression loss detected 2, and PMS2 expression loss detected none. These loss of expression events were highly specific predictors of both tumor MSI and hypermutation status, occurring almost exclusively within hypermutated and MSI-H tumors. However, a subset of less than 10% of MSI-H tumors displayed normal expression levels of these 4 genes (Table 2), indicating MMR dysfunction arising from a cause other than loss of mRNA expression in these cases.

Additional files 3, 4 and 5 display the results of Fig. 1 stratified by histological subtypes. The observations of Fig. 1 hold across each cancer’s histological subtypes.

### Hypermutated tumors share common transcriptional patterns in colon, stomach, and endometrial cancers

Approximately one third of the hypermutation or ultramutation events as measured by next-generation sequencing in TCGA (a broader set than MSI-H tumors) cannot be detected by loss of MMR gene expression. In such cases, transcriptomic events downstream of MMRd might enable detection of hypermutation independent of the expression levels of the classic MMR genes. In cancers where hypermutation has a common origin in MMRd, and possibly in CIMP, we hypothesized that hypermutated tumors would display common transcriptional patterns across tumor types. To evaluate whether broader expression patterns could predict tumor MSI and hypermutation status, we ran univariate linear models testing the association of hypermutation status with the expression levels of each gene in each of the 3 TCGA whole transcriptome RNA-Seq datasets considered.

**Fig. 2:**
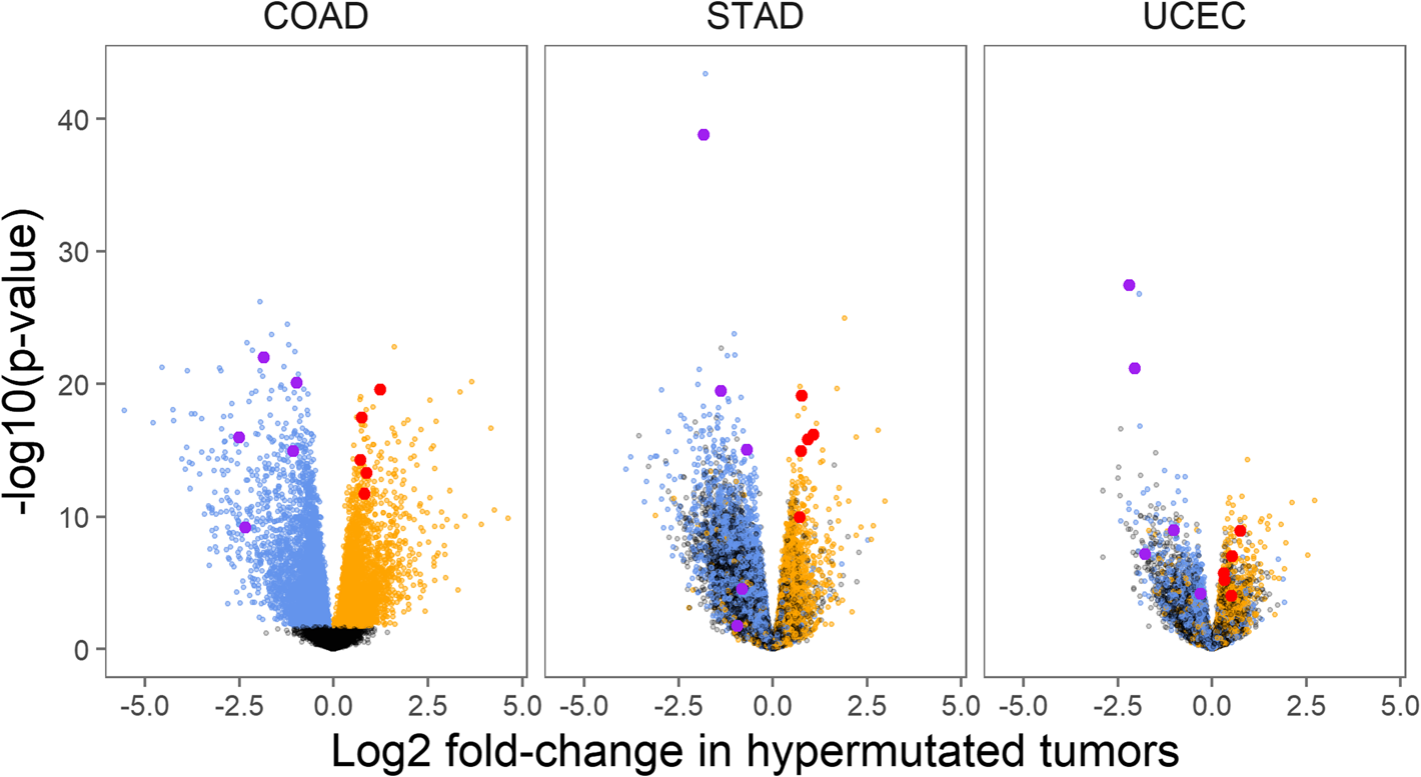
Gene expression signature of hypermutation status in TCGA dataset. Volcano plots show genes’ associations with hypermutation for colon adenocarcinoma (COAD), stomach adenocarcinoma (STAD), and uterine corpus endometrial carcinoma (UCEC). Genes with a false discovery rate (FDR) < 0.05 in COAD are colored orange and blue in all 3 panels based on the direction of their association with hypermutation in COAD. The genes used by the Hypermutation Predictor algorithm are highlighted in red (positive weights) and purple (negative weights)

Genes with highly significant associations with tumor hypermutation status were abundant: a Benjamini-Hochberg false discovery rate (FDR) < 0.05 was achieved by 7800 genes in colon adenocarcinomas, 9337 genes in stomach adenocarcinomas, and 3848 genes in endometrial carcinomas. A number of these genes behaved similarly across all 3 cancer types: 420 genes had a FDR < 0.05 and a positive association with tumor hypermutation status in all 3 datasets, and 672 genes had a FDR < 0.05 and a negative association with tumor hypermutation status in all 3 cancer types (Fig. 2). Gene sets relating to DNA replication machinery and metabolism were highly enriched for positive associations with hypermutation (Additional file 6). The results demonstrated that numerous genes display strong differential expression with tumor hypermutation status across all cancer types and suggest that a data-driven predictor of tumor hypermutation status could prove informative.

### Novel gene expression algorithms for predicting MMRd, Hypermutation, and MSI status

Based on the above observations in the TCGA dataset, separate gene expression algorithms were trained for predicting tumor MMR Loss and Hypermutation status, and then combined into a single “MSI Predictor” algorithm. The “MMR Loss” algorithm, informed by the results of Fig. 1, measures loss of tumor expression for the 4 MMR genes (MLH1, MSH2, MSH6, and PMS2). The “Hypermutation Predictor” algorithm, informed by the results of Fig. 2, uses 10 genes differentially expressed in hypermutated tumors to predict a tumor’s hypermutation status. Finally, to maximize predictive value by using all available information, the MSI Predictor algorithm combines the MMR Loss and Hypermutation Predictor scores into a single score designed to predict tumor MSI status. The derivations and calculations of these algorithms are summarized below and described in detail in Additional file 7.

### The MMR loss algorithm for calling tumor MSI status based on tumor loss of MMR gene expression

An algorithm for predicting tumor MSI status by detecting loss of expression in the four MMR genes MLH1, MSH2, MSH6 and PMS2 was developed using the TCGA datasets for the 3 tumor types known to have relatively high prevalence of MSI-H status (i.e. colon, endometrial and gastric cancers). The algorithm is based on the hypothesis that MSI-H status will occur in most instances when one or more of the MMR genes suffers severe loss of expression. It evaluates each gene for expression loss compared to the normal expression range seen in MMR proficient tumors, and it reports the magnitude of the most severe expression loss among the four genes.

### The Hypermutation predictor algorithm for calling MSI status from genes differentially expressed in hypermutated tumors

Although the MMR Loss algorithm is expected to accurately identify the majority of MSI-H tumors, it is expected to fail in tumors whose MSI-H results from mutations in the MMR genes that do not affect transcriptional levels, or from post-transcriptional regulation of the proteins. Thus, we developed an independent method for calculating MSI-H status based on differential gene expression observed between hypermutated and non-hypermutated samples in the three TCGA datasets where MSI-H status is common (Fig. 2). Based on this analysis, ten genes were selected that had strong differential expression in all three datasets, as well as large effect sizes in models fit to subsets of the data that excluded ultramutated tumors or hypermutated tumors without MMR gene expression loss.

Using the 10 selected genes, a linear predictor score was derived using methods similar to Wright et al. [28]. Table 1 details the selected genes and their weights in the Hypermutation Predictor score. A detailed description of the derivation and calculation of the Hypermutation Predictor algorithm is provided in the Additional file 7: Supplementary Methods.

**Table 1:** Algorithm weights and false discovery rates of the genes in the Hypermutation Predictor score

Gene	Weight	COAD FDR	STAD FDR	UCEC FDR
EPM2AIP1	−0.31218	2.13E-19	1.49E-35	6.80E-24
TTC30A	−0.19894	1.54E-13	5.22E-17	2.59E-07
SMAP1	−0.1835	7.96E-18	2.57E-13	0.001251
RNLS	−0.19023	2.23E-14	0.000156	4.52E-18
WNT11	−0.11515	1.52E-08	0.036791	7.02E-06
SFXN1	0.214676	1.22E-15	1.11E-16	0.000229
SREBF1	0.194835	8.58E-11	5.48E-14	8.62E-06
TYMS	0.206972	2.08E-17	2.73E-14	0.001611
EIF5AL1	0.194935	5.99E-13	2.86E-13	9.06E-05
WDR76	0.188582	4.26E-12	3.80E-09	2.67E-07

Note: *COAD* colon adenocarcinoma, *FDR* false discovery rate, *STAD* stomach adenocarcinoma, *UCEC* uterine corpus endometrial carcinoma

### The MSI predictor algorithm for calling tumor MSI status from combined information in the MMR loss and Hypermutation predictor scores

Ultimately, a single procedure was required for calling tumors’ MSI status. The MSI predictor algorithm described below combines the information in the MMR Loss and Hypermutation Predictor scores into a single score for predicting MSI status. This algorithm was designed to have two properties. First, when either the MMR Loss algorithm or the hypermutation algorithm suggests MSI-H status with high confidence, the other algorithm should not be allowed to counteract this finding. Second, when both algorithms suggest MSI-H status, the evidence they provide should be evaluated jointly to gain additional confidence in an MSI-H call. A detailed description of the MSI Predictor algorithm is included in Additional file 7.

**Fig. 3:**
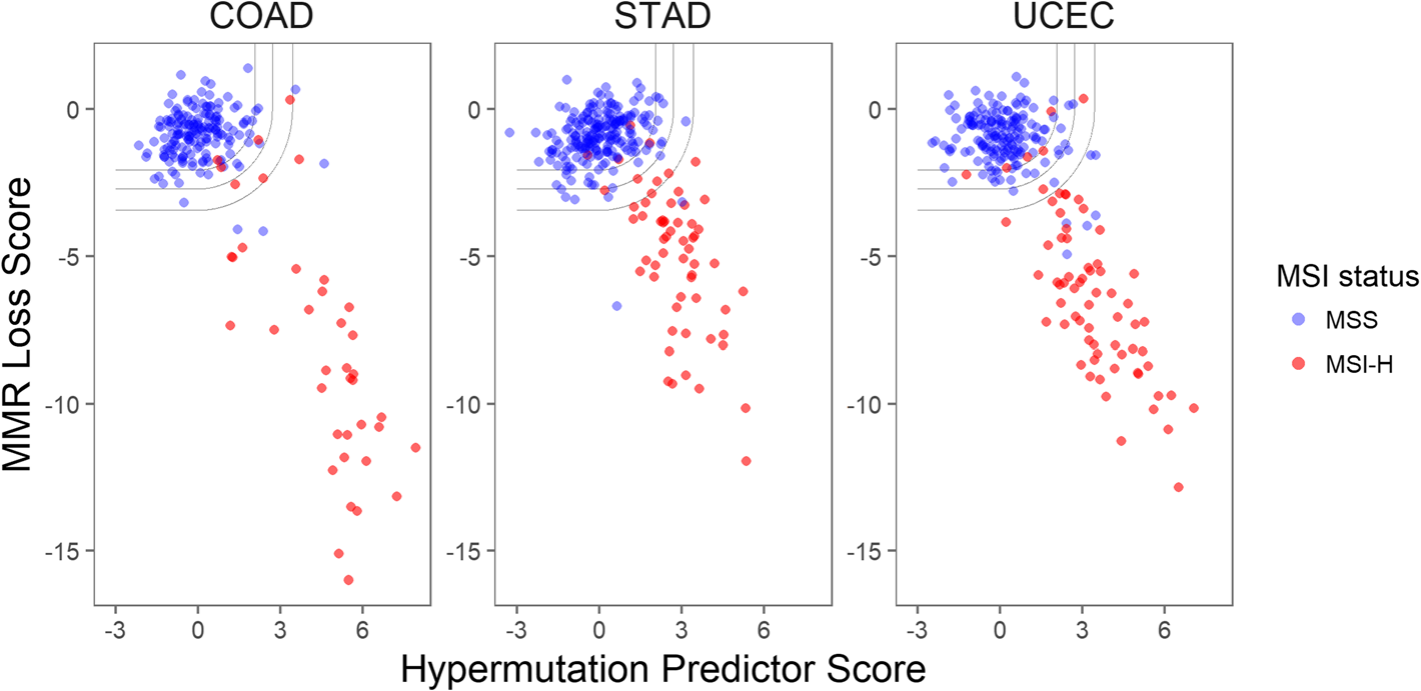
Relationship between MMR Loss score, Hypermutation Predictor score, *and MSI Predictor score.* Curved lines show the decision boundaries corresponding, from top-left to bottom-right, to microsatellite instability (MSI) Predictor score *p*-value cutoffs of 0.05, 0.01, and 0.001. Color denotes tumor MSI status

Figure 3 shows how the 3 algorithms relate to each other. Despite capturing distinct biological signals, the MMR Loss and Hypermutation Predictor scores were correlated but not redundant. And by combining the evidence from the other two algorithms, the MSI Predictor score better classified borderline samples. The curved decision boundaries shown in Fig. 3 demonstrate the algorithm’s approach to combining evidence from the MMR Loss and Hypermutation Predictor algorithms.

Additional files 8, 9 and 10 display the results of Fig. 3 stratified by histological subtypes. The observations of Fig. 3 hold across each cancer’s histological subtypes.

### Gene expression algorithms predict tumor MSI status in TCGA training datasets

We evaluated the ability of the MSI Predictor algorithm and its 2 component algorithms to predict tumor MSI status in TCGA colon adenocarcinoma (COAD), stomach adenocarcinoma (STAD), and uterine corpus endometrial carcinoma (UCEC). The MMR Loss and Hypermutation Predictor algorithms were each on their own accurate predictors of tumor MSI status, but the MSI Predictor algorithm showed higher accuracy as measured by true positive rate (TPR) and false positive rate (FPR) (Table 2).

**Table 2:** Performance of gene expression algorithms in predicting microsatellite instability

	COAD	STAD	UCEC
TPR			
MMR Loss score	0.9 (0.76–0.96)	0.92 (0.82–0.96)	0.94 (0.86–0.98)
Hypermutation Predictor score	0.74 (0.59–0.85)	0.8 (0.68–0.88)	0.94 (0.86–0.98)
MSI Predictor score	0.9 (0.76–0.96)	0.9 (0.8–0.95)	0.93 (0.84–0.97)
FPR			
MMR loss score	0.26 (0.2–0.32)	0.3 (0.24–0.36)	0.36 (0.3–0.43)
Hypermutation Predictor score	0.17 (0.12–0.23)	0.23 (0.18–0.29)	0.37 (0.31–0.43)
MSI Predictor score	0.21 (0.16–0.28)	0.25 (0.19–0.31)	0.3 (0.24–0.36)

Note: A *p*-value threshold of 0.01 was used for all gene expression algorithms. True positive rate (TPR) is the proportion of high-level microsatellite instability (MSI-H) cases detected by each algorithm. False positive rate (FPR) is the proportion of non-hypermutated cases falsely called hypermutated by the gene expression algorithms. Numbers in parentheses give 95% confidence intervals calculated by the Wilson method. *COAD* colon adenocarcinoma, *STAD* stomach adenocarcinoma, *UCEC* uterine corpus endometrial carcinoma

### Gene expression algorithms predict tumor hypermutation status in TCGA training datasets

The gene expression algorithms predicted tumor hypermutation in TCGA datasets almost as well as they predicted tumor MSI status (Table 3), though TCGA’s PCR-based MSI assay was a slightly more powerful predictor of tumor hypermutation status than gene expression.

**Table 3:** Performance of gene expression algorithms and microsatellite instability in predicting hypermutation

	COAD	STAD	UCEC
TPR			
MMR Loss score	0.77 (0.62–0.87)	0.8 (0.69–0.88)	0.73 (0.63–0.81)
Hypermutation Predictor score	0.65 (0.5–0.78)	0.74 (0.63–0.83)	0.83 (0.74–0.9)
MSI Predictor score	0.79 (0.65–0.89)	0.79 (0.67–0.87)	0.74 (0.65–0.82)
MSI status	0.86 (0.73–0.93)	0.88 (0.78–0.94)	0.74 (0.65–0.82)
FPR			
MMR loss score	0.1 (0.06–0.15)	0.11 (0.07–0.16)	0.13 (0.08–0.19)
Hypermutation Predictor score	0.02 (0.01–0.05)	0.04 (0.02–0.08)	0.12 (0.08–0.18)
MSI Predictor score	0.04 (0.02–0.08)	0.03 (0.02–0.07)	0.03 (0.01–0.07)
MSI status	0.01 (0–0.04)	0 (0–0.03)	0.01 (0–0.05)

Note: A *p*-value threshold of 0.01 was used for all gene expression algorithms. True positive rate (TPR) is the proportion of hypermutated cases detected by algorithm scores below a p-value threshold of 0.01 or by a high-level microsatellite instability (MSI-H) call. False positive rate (FPR) is the proportion of non-hypermutated cases falsely called hypermutated by gene expression algorithms of MSI-H calls. Numbers in parentheses give 95% confidence intervals calculated by the Wilson method. COAD = colon adenocarcinoma; STAD = stomach adenocarcinoma; UCEC = uterine corpus endometrial carcinoma

### Validation of tumor MSI predictor algorithm in two independent sample sets

To validate the algorithms trained in TCGA datasets, the NanoString nCounter Analysis System (NanoString Technologies, Inc., Seattle, Washington, USA) was used to profile two new sample sets for which results of the MMRd IHC assay were available. One sample set consisted of 25 MMR-proficient and 27 MMRd colorectal carcinoma samples and the second sample set was 5 MMR-proficient and 10 MMRd endometrial and neuroendocrine tumors. The endometrial and neuroendocrine samples were combined in a single analysis because of the limited sample size and because both are hormonally driven tumors. Additional files 11 and 12 contain relevant expression, immunohistochemistry, qPCR and sequencing data from these sample sets.

**Fig. 4:**
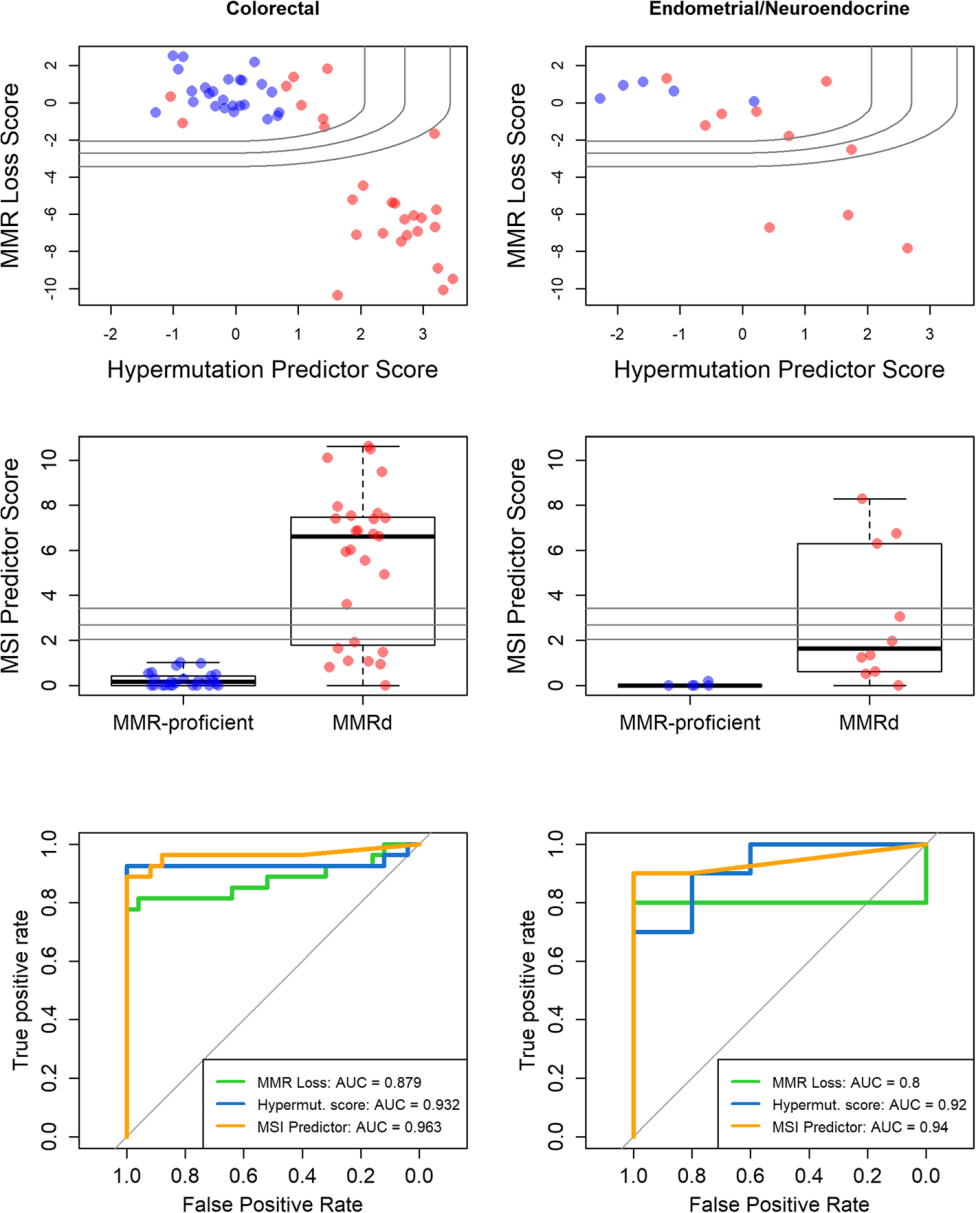
Performance of microsatellite instability (MSI) prediction algorithms in colorectal and endometrial/neuroendocrine cancer sample sets. Left column: colorectal samples; right column: endometrial/neuroendocrine samples. Blue points are mismatch repair (MMR)-proficient tumors; red points are MSI-H. Top row: the Hypermutation Predictor and MMR Loss scores are plotted against each other. Lines show the regions of the plot corresponding to p-value thresholds of 0.05, 0.01, and 0.001 on the MSI Predictor score, in which all points below the line would be called MSI-H. Middle row: values of the MSI Predictor score are plotted vs. microsatellite stable (MSS)/MSI-H status. The *p* = 0.05, 0.01, and 0.001 thresholds are indicated with horizontal lines. Bottom row: receiver operating characteristic (ROC) curves are shown for all 3 algorithms

Replicating the phenomenon seen in TCGA datasets, the validation datasets revealed loss of expression events in a majority of tumor MSI-H samples (Additional file 13). In the endometrial and neuroendocrine samples, expression losses were only observed for the MLH1 gene. PMS2 gene expression was not noticeably suppressed in 2 tumors with mutations in that gene and in 2 tumors with loss of nuclear PMS2 expression seen by IHC. In the colorectal samples, frequent MLH1 gene expression loss was observed (69% of MSI-H tumors), as was a single instance each of MSH2 and PMS2 loss. Loss of expression events occurred exclusively in MMRd tumors. The MMR Loss score, which measures the evidence for loss of expression in any of the 4 MMR genes, attained an area under the receiver operating characteristic (ROC) curve of 0.80 in endometrial/neuroendocrine samples and 0.87 in colorectal samples (Fig. 4).

The Hypermutation Predictor score, a linear combination of 10 genes, retained strong predictive performance in these independent datasets and outperformed the MMR Loss score (area under curve [AUC] = 0.902 in endometrial/neuroendocrine samples and 0.932 in colorectal samples) (Fig. 4). The MSI Predictor score added predictive power to the Hypermutation Predictor score. The majority of MMRd cases were unambiguously detected by the MSI Predictor score, and the score’s overall predictive power was very high (AUC = 0.940 in endometrial/neuroendocrine samples and 0.938 in colorectal samples).

### Association of tumor MSI status with level of anti-tumor immunity as measured by the tumor inflammation signature

**Fig. 5:**
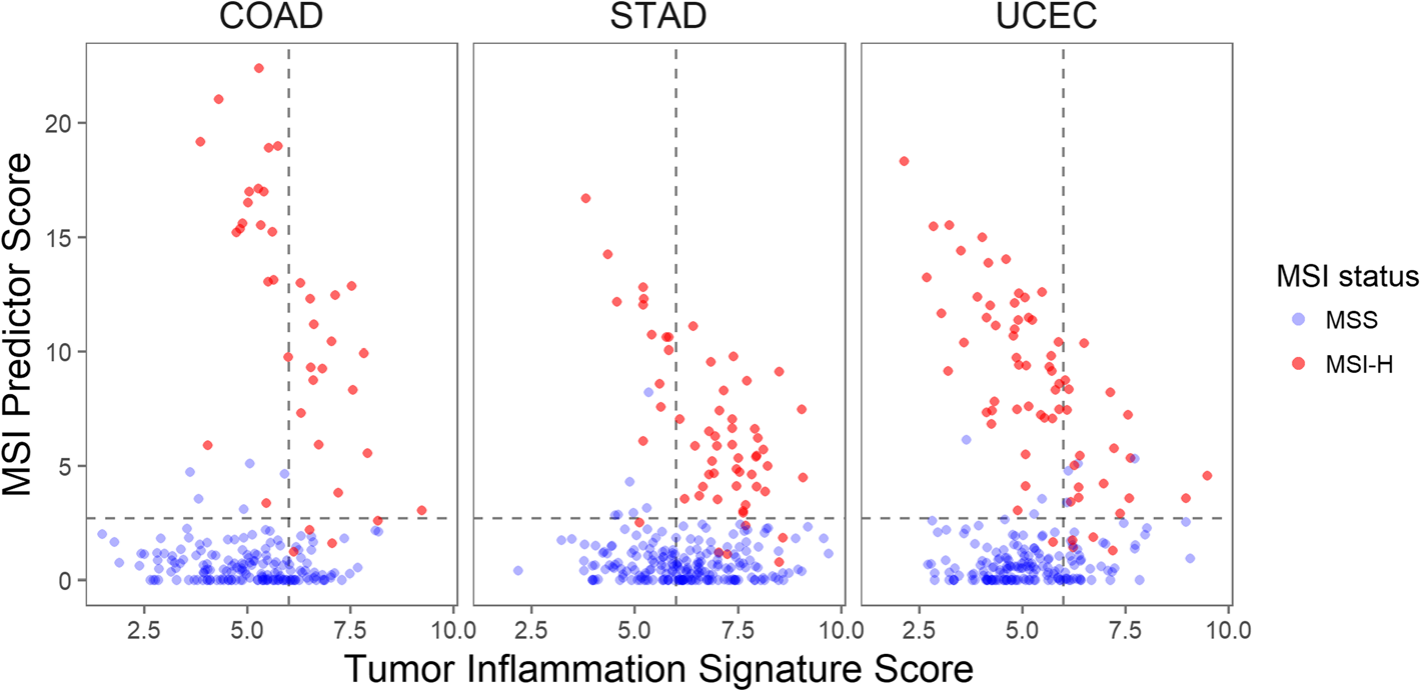
Relationship between MSI Predictor score and Tumor Inflammation Signature (TIS) in theTCG datasets). Color denotes high-level microsatellite instability (MSI-H) vs. microsatellite stable (MSS) tumors as reported in the TCGA database. Lines show cutoffs for each assay: the MSI Predictor score threshold corresponds to a *p*-value cutoff of 0.01, and the TIS score threshold is set at a level recommended by Danaher et al. 2018 [7]

The Tumor Inflammation Signature (TIS) was developed and analytically and clinically validated in the context of single agent pembrolizumab and measures the expression of 18 genes, reflecting the presence of a peripherally suppressed adaptive immune response in the tumor micro-environment [5]. The TIS is largely independent from tumor mutational burden, suggesting that an integration of these two measurements can carry improved predictive value [7]. Figure 5 uses gene expression alone to compare the genotype variable of tumor MSI status to the phenotype variable of local anti-tumor immunity, plotting the MSI Predictor score against the TIS score in the TCGA COAD, STAD, and UCEC datasets.

Together, the TIS and MSI Predictor scores measured simultaneously in the same sample identified more patients likely to benefit from checkpoint inhibition than either test alone. Across these 3 datasets, only 2 samples identified as MSI-H by standard techniques were missed by both the TIS and MSI gene expression score.

Additional files 14, 15 and 16 display the results of Fig. 5 stratified by histological subtype. The observations of Fig. 5 hold across each cancer’s histological subtypes.

## Discussion

This study demonstrated that gene expression can be used to identify MSI-H tumors with both high sensitivity and specificity. This discovery opens the possibility of using gene expression profiling to identify multiple orthogonal biomarkers of checkpoint inhibitor efficacy in a single assay, thereby improving the ability to identify the best treatment option for every patient. Indeed, this possibility was forecasted by the work of Cristescu et al. [29], who reported the correlation of the TIS measured on the nCounter platform (which they call GEP) and tumor mutation burden as measured by whole exome sequencing to predict response to anti-PD1 therapy. In this work, we demonstrate the practical advantages to measuring both anti-tumor immune activity and MSI status using a single test. Rather than using multiple tissue samples and potentially sending those out to multiple laboratories for analysis, combining these two measurements into a single assay allows for conservation of biological material and simplification of personalized treatment decisions.

This study has some notable limitations, which need to be considered for appropriate data interpretation. First, because the Hypermutation Predictor algorithm was trained using TCGA samples, its predictive performance in TCGA (Tables 2 and 3), as well as the performance of the MSI Predictor algorithm, may be over-estimated. In contrast, the MMR Loss algorithm was developed using a minimal training procedure that only required estimates of the mean and interquartile range of each gene in non-hypermutated samples; as such, this algorithm’s performance in TCGA datasets is more likely to be representative of what would be expected in an independent dataset.

Second, one assumption underlying the training of the algorithms was that the standard deviation (SD) in gene expression levels for a gene in the TCGA RNAseq dataset would be the same in NanoString data; however, the NanoString validation results contradicted this assumption and achieved sub-optimal prediction as a result. Namely, examining the top row of Fig. 4, it appears that moving the score contours/ decision boundaries left would capture more MMRd samples while incurring no false positives. These suboptimal decision boundaries of the Hypermutation Predictor score appear to result from a lower SD in the validation MSS samples than in TCGA MSS samples. If the Hypermutation Predictor score’s SD in MSS samples were to be estimated anew in these datasets, it would shift the score contours/ decision boundaries left and thereby achieve even better prediction. Because the MSI Predictor score as implemented in the independent datasets used the pre-defined SD sestimates from TCGA datasets, it underutilized the Hypermutation Predictor score and was potentially unnecessarily conservative as a result. The reason for the narrower distribution of Hypermutation Predictor scores in MSS samples in NanoString data is unclear. It could result from more precise gene expression measurements or from some unknown difference in the studies’ sample preparation methods or clinical populations or could be a spurious observation resulting from the uncontrolled datasets.

Unexpectedly, MSI predictor scores were inversely correlated with TIS in true MSI-H samples. One possible explanation for this phenomenon is that in inflamed tumors, highly abundant immune cells contribute background expression of MLH1 and other MSI signature genes, clouding the otherwise clear signal of the tumor cells’ mRNA. Importantly, nearly all MSI-H tumors missed by the MSI gene expression score had high TIS scores, and therefore these tumors’ potential to respond to checkpoint inhibitors would be identified based on that variable alone.

In summary and despite the above limitations, this work shows the potential for gene expression as a MSI status assay; however, to translate this observation to the clinical setting, additional studies will be needed to refine the MSI Predictor score and develop a locked algorithm that can be applied prospectively to a single sample. These findings should have broad applicability in gene expression studies of cancer types where MSI occurs. We propose that tumor antigenicity, as measured by MSI, and immune response, as measured by inflammation status, should together form the foundation of any analysis of immunotherapy in solid tumors. Because these variables are non-redundant, they promise to offer superior prediction together than either can alone. Responders missed by one of these variables may often be identified by the other. To more optimally guide treatment choices, drug efficacy should be evaluated separately in MSI-H/TIS-high, MSI-H/TIS-low, MSS/TIS-high, and MSS/TIS-low subsets.

Finally, these methods for developing gene signatures of tumor antigenicity may have utility beyond MMRd. This first work in the space focuses on MSI-H tumors because they are accompanied by profound changes in gene expression and because the clinical utility of MSI-H detection has been demonstrated by the recent approval of pembrolizumab and nivolumab in MSI-H tumors with a postmarketing commitment to develop diagnostic assays. Tumor antigenicity arising from other sources will likely be reflected in the transcriptome in different ways. Multiple other DNA damage repair (DDR) pathways exist and are frequently dysregulated in tumors, often by gene silencing events such as loss of heterozygosity or epigenetic silencing [30], rendering them potentially detectable by gene expression profiling. For example, a transcriptional signature of homologous repair deficiency (HRD) has been reported [31], and HRD has been associated with increased immune infiltration and expression of immune checkpoints, but efficacy of immune checkpoint blockade in HRD tumors has not been established yet (reviewed by Mouw et al. [32]). In tumor types where antigenicity arises from variable mutagen exposure rather than intrinsic tumor biology, the path to a gene expression surrogate measurement is less clear, though still an active area of investigation.

Multiple gene expression assays that report status of specific DDR pathways could each be used in combination with TIS to potentially identify additional patient populations that may respond to immunotherapy checkpoint blockade beyond the indications where MMRd/MSI is the predominant form of DDR deficiency. Furthermore, assays which characterize DDR and TIS status simultaneously could be deployed to appropriately select patients for target combination therapies of DDR targeting agents with immune checkpoint blockade in clinical settings where monotherapy is insufficient. Gene expression profiling of tumor intrinsic DNA repair pathways in combination with profiling of immune activity within the tumor has the potential to further guide the development and deployment of immunotherapies to patient populations most likely to respond and increase their potential for positive clinical benefit.

## Additional files

Additional file 1: Code and data for training analysis in TCGA data. The R code and data used in the TCGA analyses are included in this zip file. Code executes in the directory in which it is placed. (TIFF 10912 kb)

Additional file 2: Code and data for the validation dataset analyses. The R code and data used in the colorectal and endometrial/neuroendocrine validation analyses are included in this zip file. Code executes in the directory in which it is placed. (CSV 2 kb)

Additional file 3: Expression of tumor mismatch repair genes versus tumor mutation burden across histological subtypes of TCGA COAD datasets. Each column shows data from a single histological subtype in TCGA COAD dataset, and each row shows data from a single gene. Color denotes tumor microsatellite instability (MSI) status. (TIFF 21093 kb)

Additional file 4: Expression of tumor mismatch repair genes versus tumor mutation burden across histological subtypes of TCGA STAD dataset. Each column shows data from a single histological subtype in TCGA STAD dataset, and each row shows data from a single gene. Color denotes tumor microsatellite instability (MSI) status. (TIFF 29531 kb)

Additional file 5: Expression of tumor mismatch repair genes versus tumor mutation burden across histological subtypes of TCGA UCEC dataset. Each column shows data from a single histological subtype in TCGA UCEC dataset, and each row shows data from a single gene. Color denotes tumor microsatellite instability (MSI) status. (TIFF 21093 kb)

Additional file 6: Gene set enrichment results. For all KEGG, Reactome, and Biocarta gene sets, the proportion of genes that are up- and down-regulated with a FDR < 0.05. (CSV 50 kb)

Additional file 7: Supplementary material regarding algorithm development and validation. (DOCX 30 kb)

Additional file 8: Mismatch repair (MMR) Loss and Hypermutation Predictor scores plotted against each other across histological subtypes in TCGA COAD dataset. Curved lines show the decision boundaries corresponding, from top-left to bottom-right, to microsatellite instability (MSI) Predictor score *p*-value cutoffs of 0.05, 0.01, and 0.001. Each panel shows results from a distinct subtype of TCGA COAD dataset. Color denotes tumor MSI status. (TIFF 8437 kb)

Additional file 9: Mismatch repair (MMR) Loss and Hypermutation Predictor scores plotted against each other across histological subtypes in TCGA STAD dataset. Curved lines show the decision boundaries corresponding, from top-left to bottom-right, to microsatellite instability (MSI) Predictor score *p*-value cutoffs of 0.05, 0.01, and 0.001. Each panel shows results from a distinct subtype of TCGA STAD dataset. Color denotes tumor MSI status. (TIFF 10912 kb)

Additional file 10: Mismatch repair (MMR) Loss and Hypermutation Predictor scores plotted against each other across histological subtypes in TCGA UCEC dataset. Curved lines show the decision boundaries corresponding, from top-left to bottom-right, to microsatellite instability (MSI) Predictor score *p*-value cutoffs of 0.05, 0.01, and 0.001. Each panel shows results from a distinct subtype of TCGA UCEC dataset. Color denotes tumor MSI status. (TIFF 8230 kb)

Additional file 11: Detailed summary of endometrial and neuroendocrine tumor samples used in algorithm validation studies. For the samples from the endometrial/neuroendocrine tumor validation dataset, relevant measurements from the NanoString platform, from immunohistochemistry, from qPCR, and from sequencing are provided. (CSV 2 kb)

Additional file 12: Detailed summary of colorectal tumor samples used in algorithm validation studies. For the samples from the colorectal tumor validation dataset, relevant measurements from the NanoString platform, from immunohistochemistry, and from the qPCR MSI assay are provided. (CSV 3 kb)

Additional file 13: MMR genes vs. MSI-high status in validation datasets. Normalized expression levels of the MMR genes MLH1, MSH2, MSH6, and PSM2 are plotted against MSI-high status in the colorectal and endometrial/neuroendocrine validation datasets. Solid green lines show the mean MMR-proficient expression as estimated using Gaussian mixture models without reference to MSI-high status; dashed green lines show the lower 95% quantile of expression in MMR-proficient samples derived from this mixture model mean and the SD calculated in TCGA. (TIFF 12920 kb)

Additional file 14: Microsatellite instability (MSI) predictor signature plotted against Tumor Inflammation Signature (TIS) across histological subtypes of TCGA COAD dataset. Each panel shows a distinct histological subtype of TCGA COAD dataset. Color denotes high-level microsatellite instability (MSI-H) vs. microsatellite stable (MSS) tumors as determined by conventional tests. Lines show cutoffs for each assay: the MSI Predictor score threshold corresponds to a *p*-value cutoff of 0.01, and the TIS score threshold is set at a level recommended by Danaher et al. 2018. (TIFF 8230 kb)

Additional file 15: Microsatellite instability (MSI) predictor signature plotted against Tumor Inflammation Signature (TIS) across histological subtypes of TCGA STAD dataset. Each panel shows a distinct histological subtype of TCGA STAD dataset. Color denotes high-level microsatellite instability (MSI-H) vs. microsatellite stable (MSS) tumors as determined by conventional tests. Lines show cutoffs for each assay: the MSI Predictor score threshold corresponds to a p-value cutoff of 0.01, and the TIS score threshold is set at a level recommended by Danaher et al. 2018. (TIFF 24700 kb)

Additional file 16: Microsatellite instability (MSI) predictor signature plotted against Tumor Inflammation Signature (TIS) across histological subtypes of TCGA UCEC dataset. Each panel shows a distinct histological subtype of TCGA UCEC dataset. Color denotes high-level microsatellite instability (MSI-H) vs. microsatellite stable (MSS) tumors as determined by conventional tests. Lines show cutoffs for each assay: the MSI Predictor score threshold corresponds to a *p*-value cutoff of 0.01, and the TIS score threshold is set at a level recommended by Danaher et al. 2018. (TIFF 8230 kb)

## Abbreviations

AUC: area under curve
CIMP: CpG island methylator phenotype
COAD: colon adenocarcinoma
DDR: DNA damage repair
DNA: deoxyribonucleic acid
FFPE: formalin-fixed paraffin-embedded
FPR: false positive rate
HPS: Hypermutation Predictor score
HRD: homologous repair deficiency
IHC: immunohistochemistry
MLS: MMR Loss score
MMR: mismatch repair
MMRd: mismatch repair deficient
mRNA: messenger ribonucleic acid
MSI: microsatellite instability
MSI-H: high-level microsatellite instability
MSS: microsatellite stable
PCR: polymerase chain reaction
RNA: ribonucleic acid
ROC: receiver operating characteristic
SD: standard deviation
STAD: stomach adenocarcinoma
TCGA: The Cancer Genome Atlas
TIS: Tumor Inflammation Signature
TPR: true positive rate
UCEC: uterine corpus endometrial carcinoma
