# Correction to: A gene expression assay for simultaneous measurement of microsatellite instability and anti-tumor immune activity

**DOI:** 10.1186/s40425-019-0560-x

**Published:** 2019-03-15

**Authors:** Patrick Danaher, Sarah Warren, Su Fey Ong, Nathan Elliott, Alessandra Cesano, Sean Ferree

**Affiliations:** NanoString Technologies®, Inc, 530 Fairview Ave. N, Seattle, WA 98109 USA

**Correction to: Journal for ImmunoTherapy of Cancer (2019) 7:15**

**https://doi.org/10.1186/s40425-018-0472-1**

Following publication of the original article [1], the author reported the additional files have been published in the wrong format.

The correct files (Additional files 1 and 2) are attached to this Erratum.

The publisher apologizes for any inconvenience caused by this error.

## Additional files

Additional file 1: Code and data for training analysis in TCGA data. The R code and data used in the TCGA analyses are included in this zip file. Code executes in the directory in which it is placed. (ZIP 120895 kb)

Additional file 2: Code and data for the validation dataset analyses. The R code and data used in the colorectal and endometrial/neuroendocrine validation analyses are included in this zip file. Code executes in the directory in which it is placed. (ZIP 456 kb)
