# Supplementary figures and images for: A gene expression assay for simultaneous measurement of microsatellite instability and anti-tumor immune activity
Source: J Immunother Cancer. 2019 Jan 21;7:15. doi: 10.1186/s40425-018-0472-1 (PMC6341623; doi:10.1186/s40425-018-0472-1)

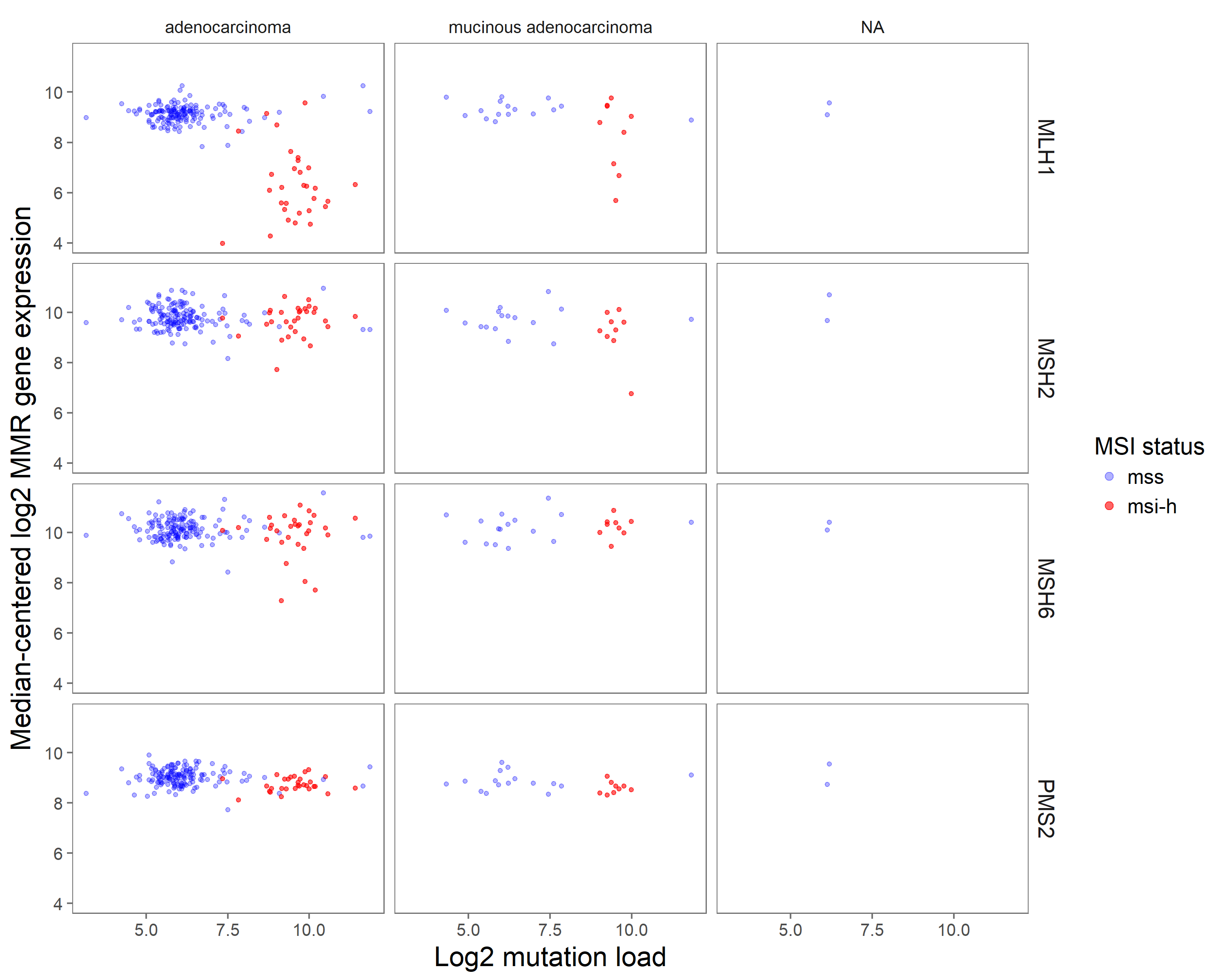

Supplement: Supplementary file 3 — Expression of tumor mismatch repair genes versus tumor mutation burden across histological subtypes of TCGA COAD datasets. Each column shows data from a single histological subtype in TCGA COAD dataset, and each row shows data from a single gene. Color denotes tumor microsatellite instability (MSI) status. (TIFF 21093 kb) [file 40425_2018_472_MOESM3_ESM.tiff]

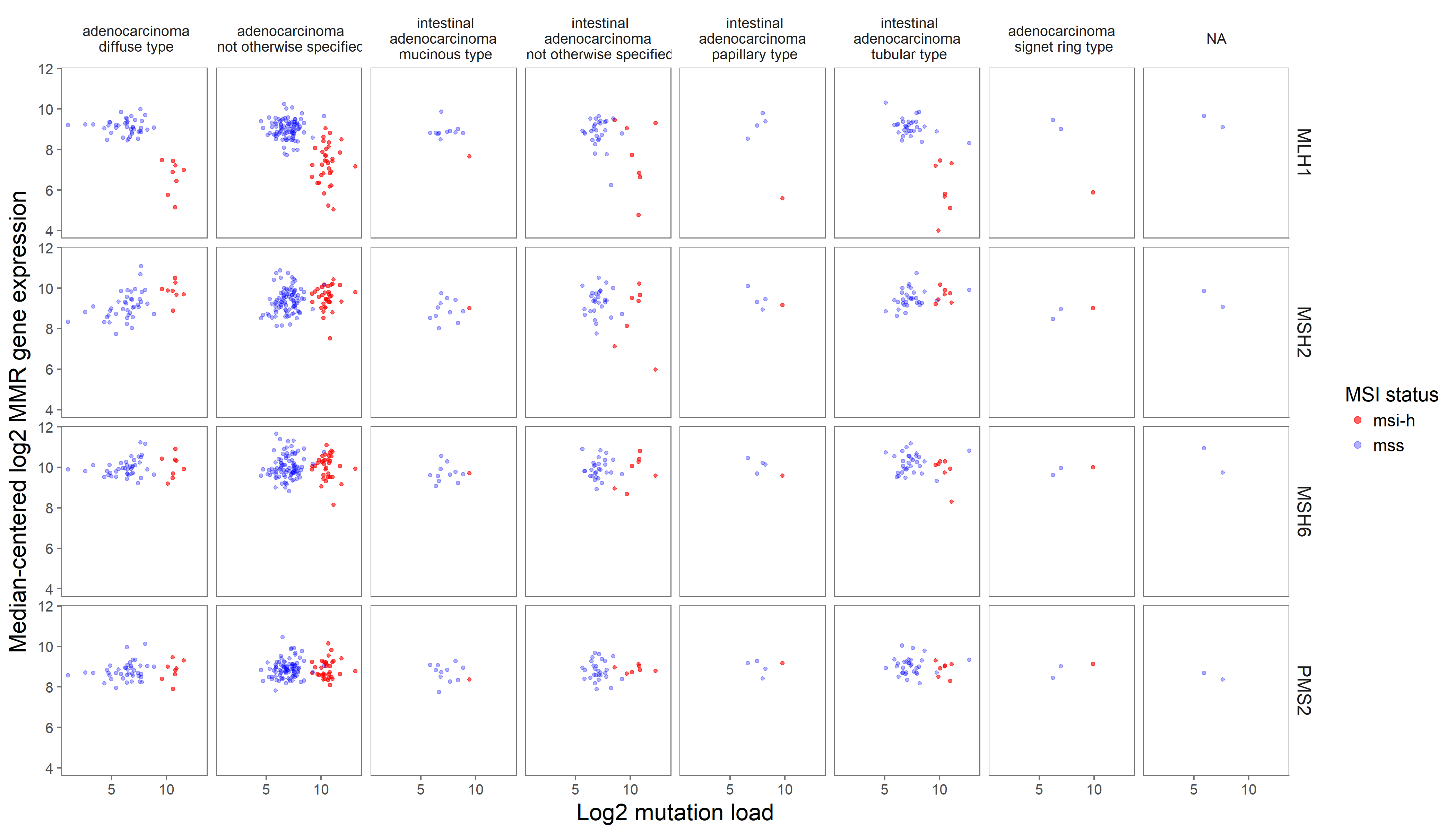

Supplement: Supplementary file 4 — Expression of tumor mismatch repair genes versus tumor mutation burden across histological subtypes of TCGA STAD dataset. Each column shows data from a single histological subtype in TCGA STAD dataset, and each row shows data from a single gene. Color denotes tumor microsatellite instability (MSI) status. (TIFF 29531 kb) [file 40425_2018_472_MOESM4_ESM.tiff]

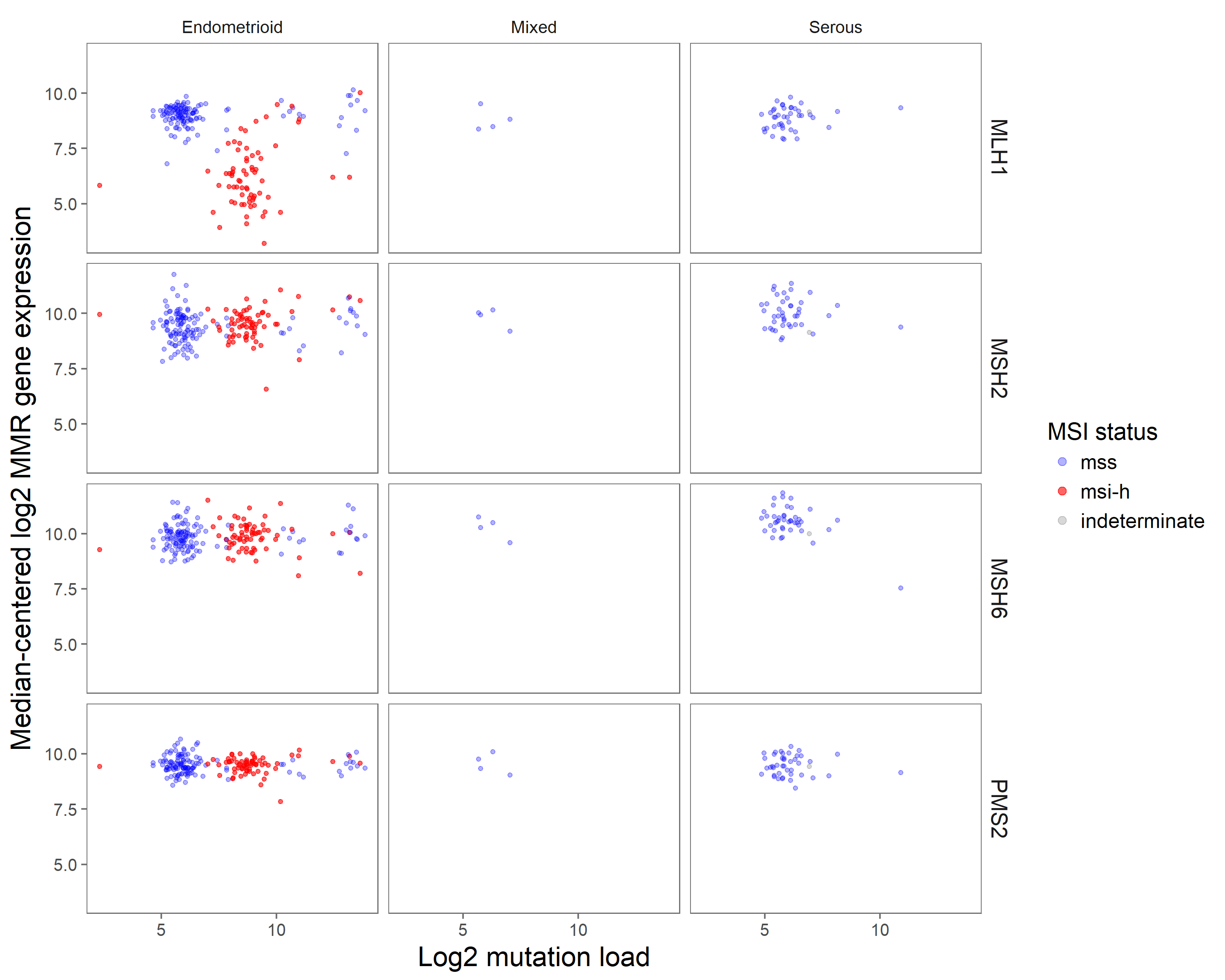

Supplement: Supplementary file 5 — Expression of tumor mismatch repair genes versus tumor mutation burden across histological subtypes of TCGA UCEC dataset. Each column shows data from a single histological subtype in TCGA UCEC dataset, and each row shows data from a single gene. Color denotes tumor microsatellite instability (MSI) status. (TIFF 21093 kb) [file 40425_2018_472_MOESM5_ESM.tiff]

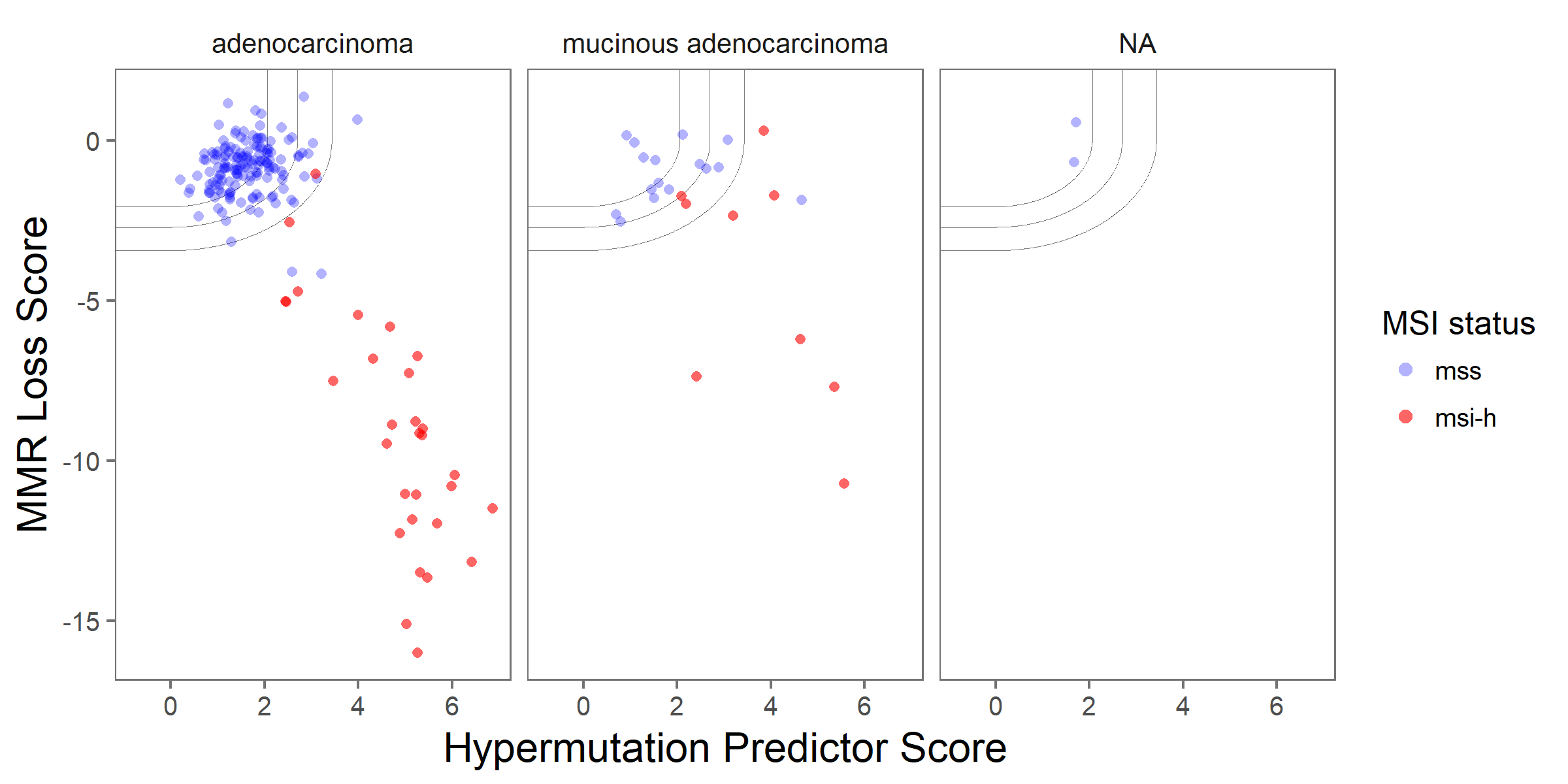

Supplement: Supplementary file 8 — Mismatch repair (MMR) Loss and Hypermutation Predictor scores plotted against each other across histological subtypes in TCGA COAD dataset. Curved lines show the decision boundaries corresponding, from top-left to bottom-right, to microsatellite instability (MSI) Predictor score p-value cutoffs of 0.05, 0.01, and 0.001. Each panel shows results from a distinct subtype of TCGA COAD dataset. Color denotes tumor MSI status. (TIFF 8437 kb) [file 40425_2018_472_MOESM8_ESM.tiff]

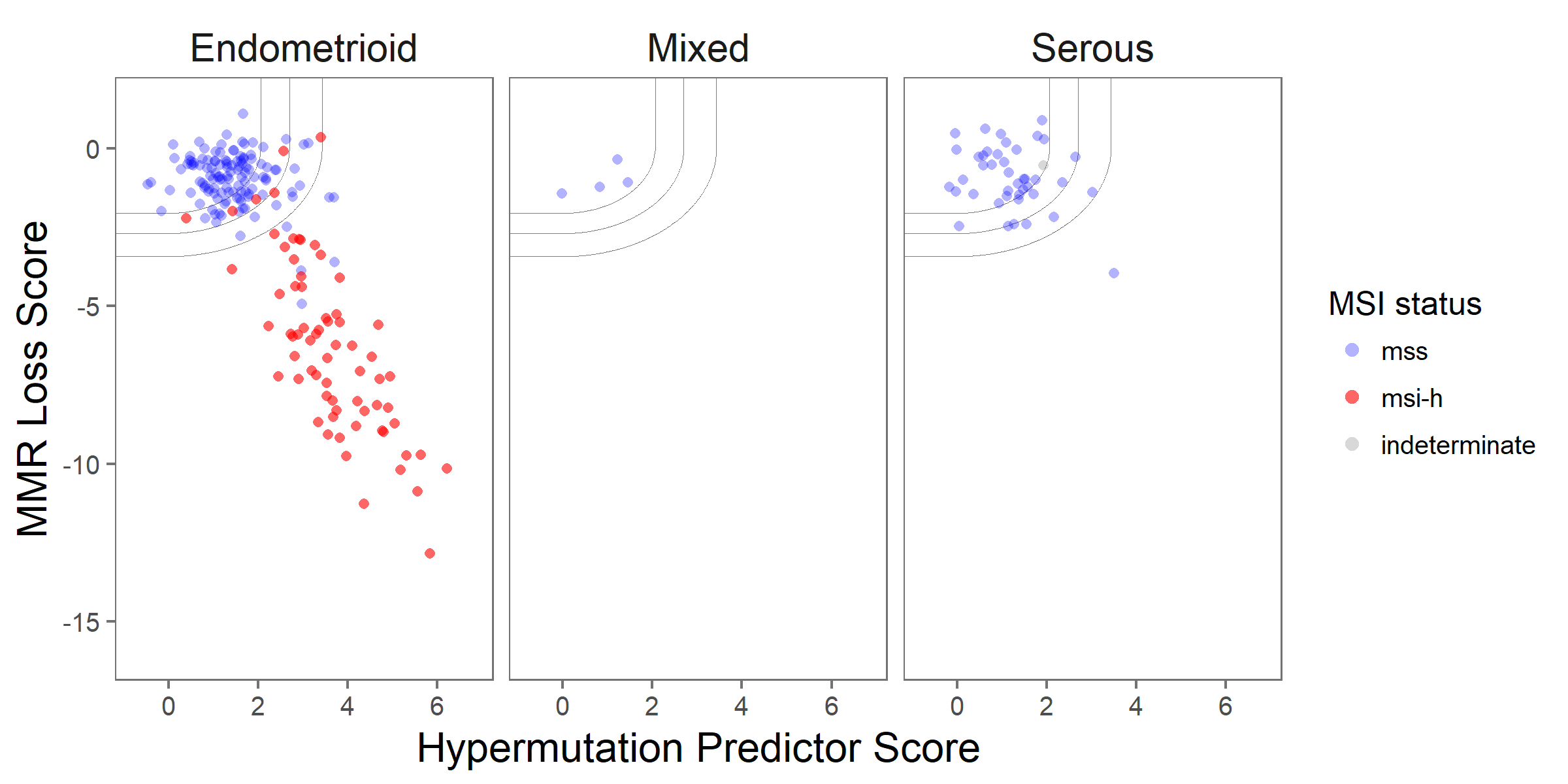

Supplement: Supplementary file 10 — Mismatch repair (MMR) Loss and Hypermutation Predictor scores plotted against each other across histological subtypes in TCGA UCEC dataset. Curved lines show the decision boundaries corresponding, from top-left to bottom-right, to microsatellite instability (MSI) Predictor score p-value cutoffs of 0.05, 0.01, and 0.001. Each panel shows results from a distinct subtype of TCGA UCEC dataset. Color denotes tumor MSI status. (TIFF 8230 kb) [file 40425_2018_472_MOESM10_ESM.tiff]

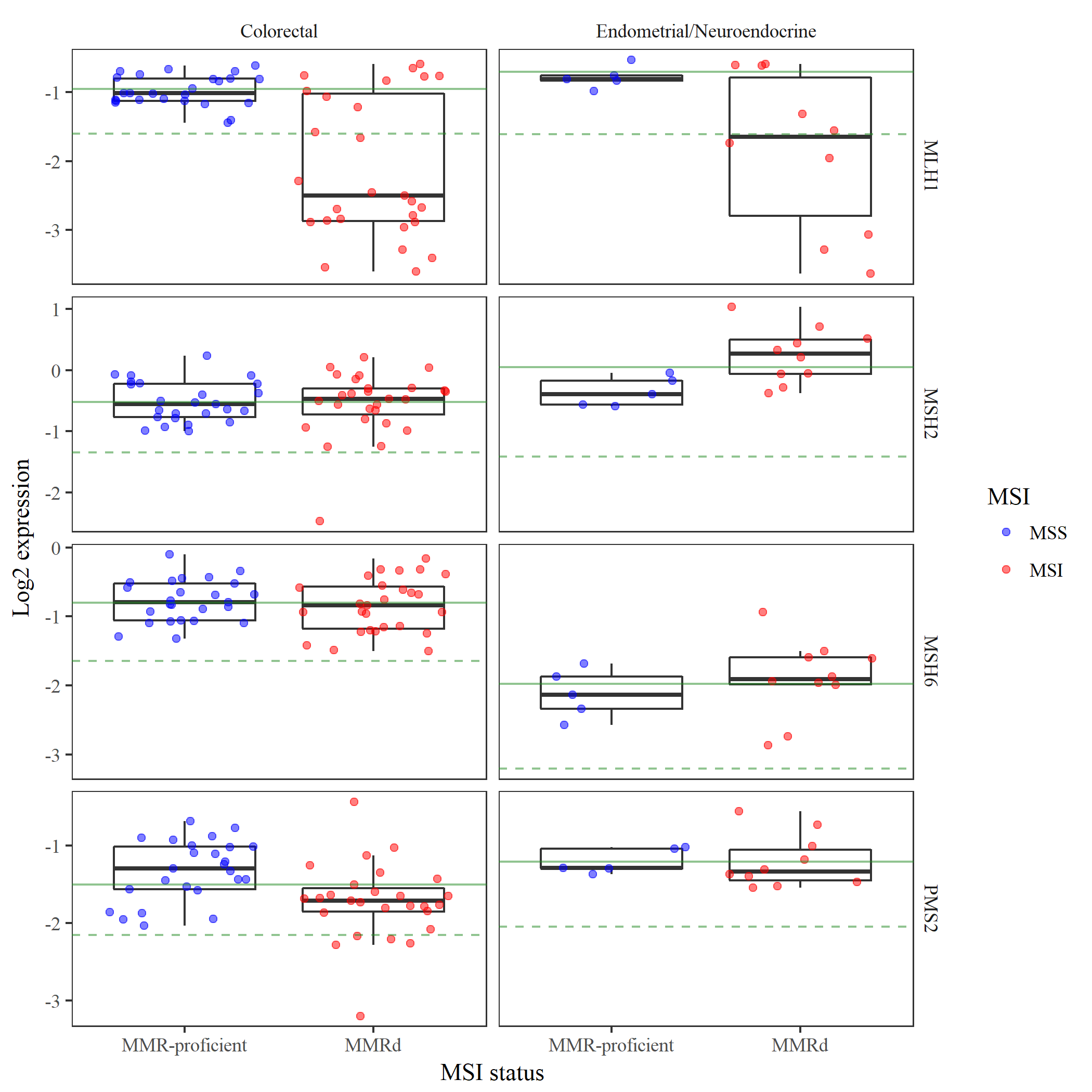

Supplement: Supplementary file 13 — MMR genes vs. MSI-high status in validation datasets. Normalized expression levels of the MMR genes MLH1, MSH2, MSH6, and PSM2 are plotted against MSI-high status in the colorectal and endometrial/neuroendocrine validation datasets. Solid green lines show the mean MMR-proficient expression as estimated using Gaussian mixture models without reference to MSI-high status; dashed green lines show the lower 95% quantile of expression in MMR-proficient samples derived from this mixture model mean and the SD calculated in TCGA. (TIFF 12920 kb) [file 40425_2018_472_MOESM13_ESM.tiff]

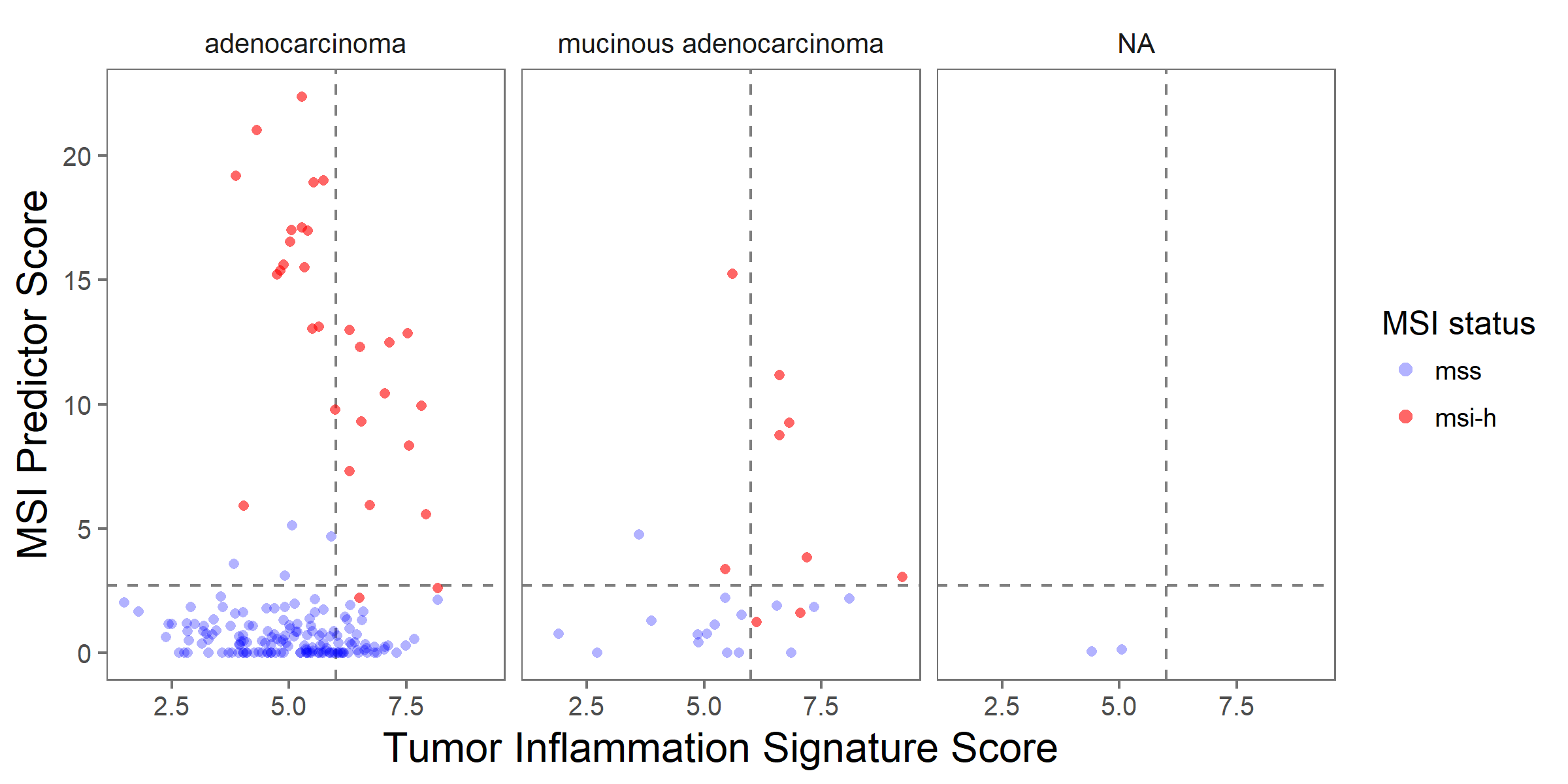

Supplement: Supplementary file 14 — Microsatellite instability (MSI) predictor signature plotted against Tumor Inflammation Signature (TIS) across histological subtypes of TCGA COAD dataset. Each panel shows a distinct histological subtype of TCGA COAD dataset. Color denotes high-level microsatellite instability (MSI-H) vs. microsatellite stable (MSS) tumors as determined by conventional tests. Lines show cutoffs for each assay: the MSI Predictor score threshold corresponds to a p-value cutoff of 0.01, and the TIS score threshold is set at a level recommended by Danaher et al. 2018. (TIFF 8230 kb) [file 40425_2018_472_MOESM14_ESM.tiff]

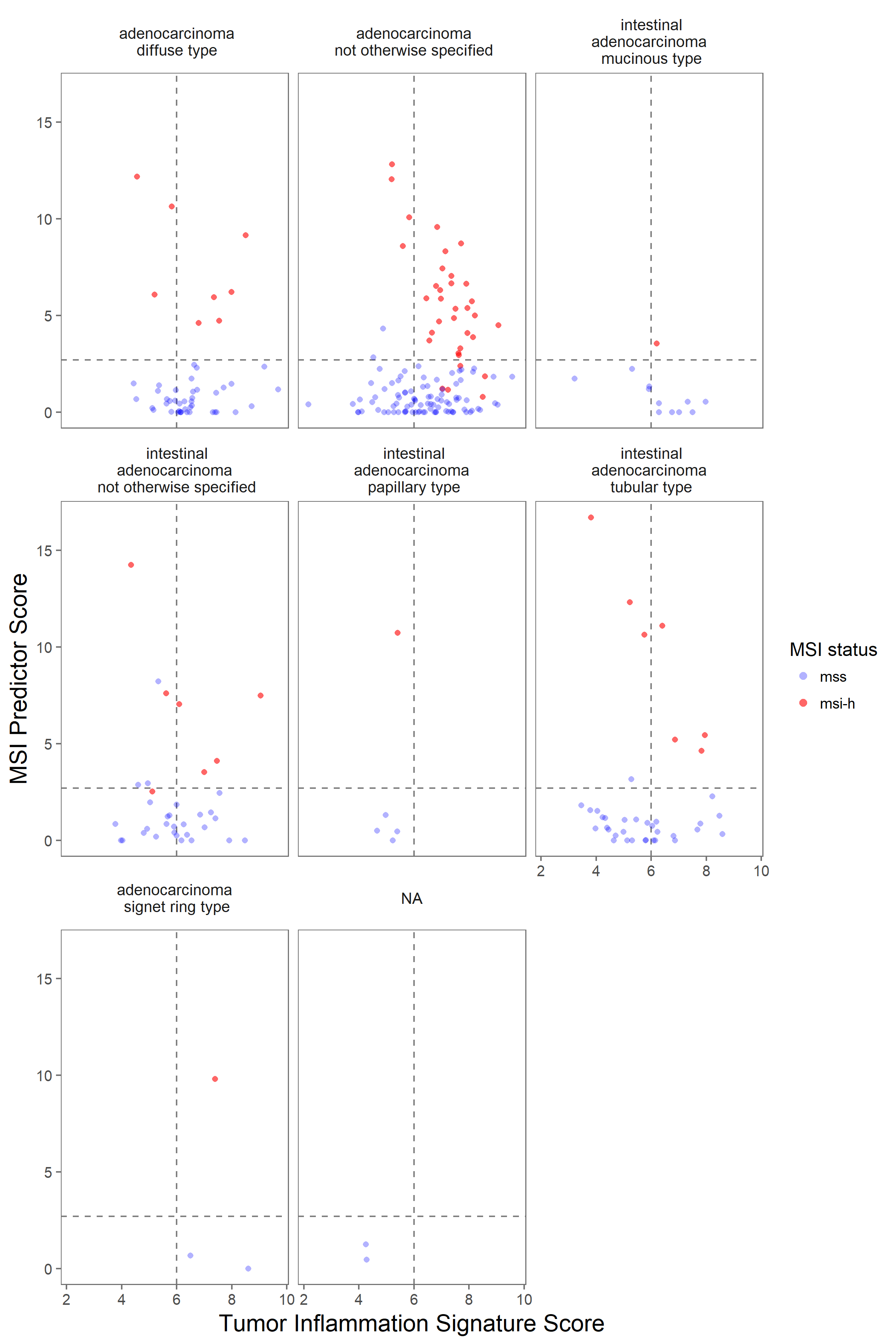

Supplement: Supplementary file 15 — Microsatellite instability (MSI) predictor signature plotted against Tumor Inflammation Signature (TIS) across histological subtypes of TCGA STAD dataset. Each panel shows a distinct histological subtype of TCGA STAD dataset. Color denotes high-level microsatellite instability (MSI-H) vs. microsatellite stable (MSS) tumors as determined by conventional tests. Lines show cutoffs for each assay: the MSI Predictor score threshold corresponds to a p-value cutoff of 0.01, and the TIS score threshold is set at a level recommended by Danaher et al. 2018. (TIFF 24700 kb) [file 40425_2018_472_MOESM15_ESM.tiff]

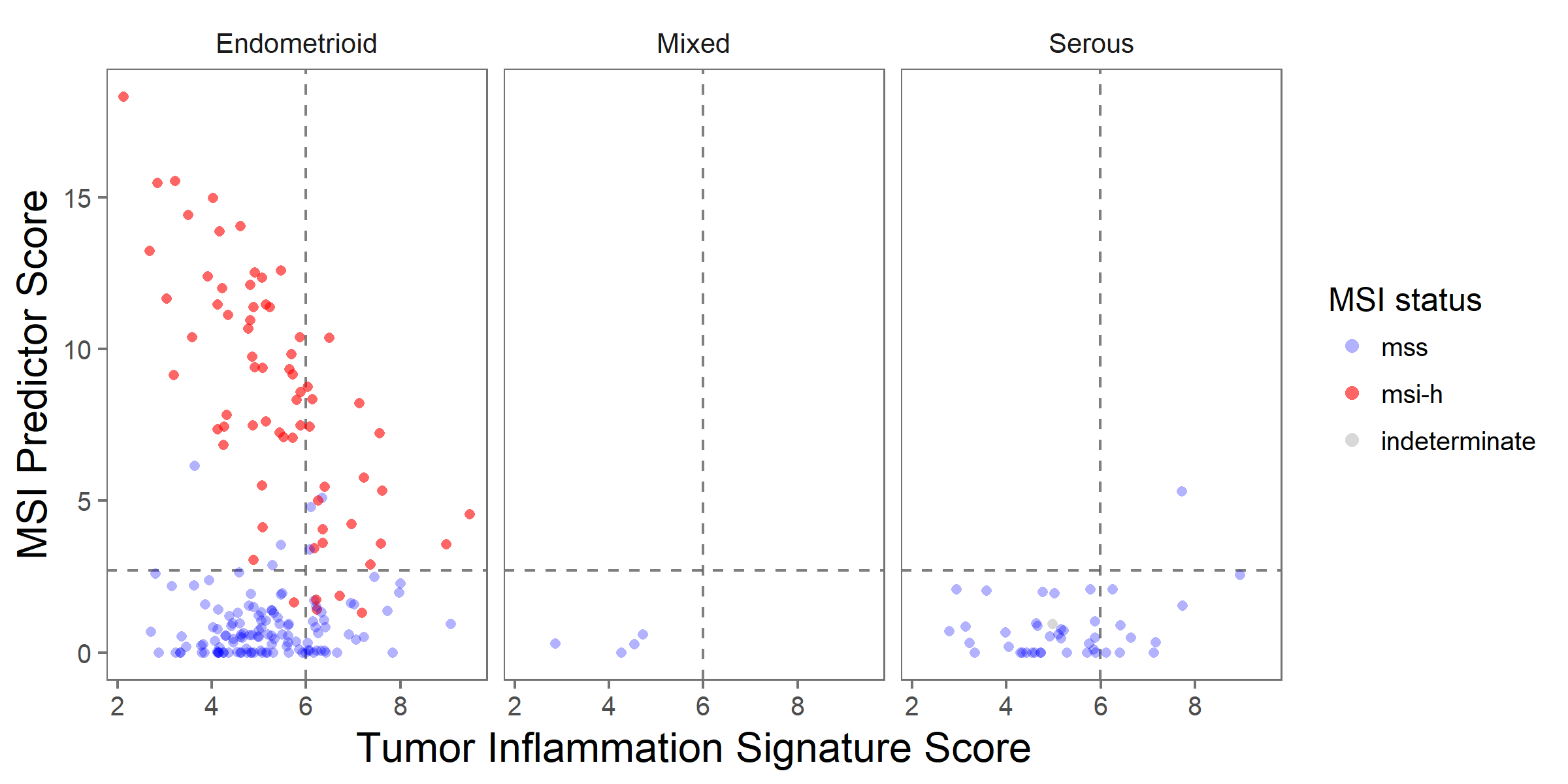

Supplement: Supplementary file 16 — Microsatellite instability (MSI) predictor signature plotted against Tumor Inflammation Signature (TIS) across histological subtypes of TCGA UCEC dataset. Each panel shows a distinct histological subtype of TCGA UCEC dataset. Color denotes high-level microsatellite instability (MSI-H) vs. microsatellite stable (MSS) tumors as determined by conventional tests. Lines show cutoffs for each assay: the MSI Predictor score threshold corresponds to a p-value cutoff of 0.01, and the TIS score threshold is set at a level recommended by Danaher et al. 2018. (TIFF 8230 kb) [file 40425_2018_472_MOESM16_ESM.tiff]
