# Supplementary material for: A gene expression assay for simultaneous measurement of microsatellite instability and anti-tumor immune activity
Source: J Immunother Cancer. 2019 Jan 21;7:15. doi: 10.1186/s40425-018-0472-1 (PMC6341623; doi:10.1186/s40425-018-0472-1)
Supplement: Supplementary file 7 — Supplementary material regarding algorithm development and validation. (DOCX 30 kb) [file 40425_2018_472_MOESM7_ESM.docx]

# Additional file 1: Supplementary Material

***Supplementary methods***

*Development of the MMR-loss algorithm*

The goal of the MMR Loss algorithm is to detect loss of expression of any of the four MMR genes MLH1, MSH2, MSH6 and PMS2. To do so, it compares each gene to the expected expression range found within MSS tumors and reports the most extreme of the four genes’ underexpression. Here we describe in detail the derivation of this procedure.

To quantify relative expression of the four MMR genes within a given sample versus the population of MSS tumors, we must estimate the mean and SD of their expression in MSS tumors. In our TCGA algorithm training exercise, we estimate these parameters directly, taking each gene’s empirical mean and SD within the MMS cases of each separate dataset. The resulting SD estimates are reported in Supplementary Table 1; we do not report the genes’ mean estimates as they are not applicable outside of TCGA database.

Estimation of the four genes’ mean parameters in subsequent datasets requires a different approach. Genes’ mean expression values will vary across gene expression platforms, normalization techniques, and experiment batches. Therefore, the MMR genes’ means in MSS samples must be estimated anew when deploying this algorithm on a dataset generated under different conditions than TCGA database. To ensure an unbiased procedure in our validation datasets, this mean parameter was estimated without reference to known tumor mutation or MSI status by fitting a Gaussian mixture model with 2 clusters and taking the mean of the higher cluster. In future datasets where MSI-H incidence is expected to be low, simply taking each gene’s median across all samples would achieve a reasonable estimate of its mean expression in MSS samples. If this algorithm were to be applied in a locked assay, each gene’s mean in MSS samples could be estimated directly and fixed thereafter.

Our approach to translating the MMR genes’ SD parameters across datasets was simpler. We assumed that the SD of a gene’s log-scale expression should have been largely platform-agnostic, as platform effects are generally well-modelled as unique scaling factors applied to each gene, amounting to additive constants on the log-scale. This assumption allowed initial estimation of this parameter in TCGA datasets and subsequent use in future datasets without further calibration. In practice, this assumption did not hold perfectly between RNASeq and NanoString data, with some genes displaying lower SDs in NanoString data than in TCGA datasets.

Once each gene’s mean and SD in MSS samples have been estimated, the algorithm proceeds in a straightforward manner to report the most extreme expression loss. Each gene is Z-scored and the minimum of the 4 Z-scores is taken for each sample. To place the score on a familiar scale, this minimum Z score is then rescaled by the theoretical mean and SD of the minimum of 4 standard normal random variables, attaining a final “MMR Loss” score with a mean of 0 and SD of 1 in non-hypermutated samples. A concise description of the procedure for calculating MMR Loss score is provided in Additional file 1.

*Development of the Hypermutation Predictor algorithm*

The Hypermutation Predictor algorithm was designed to return high scores for hypermutated tumors and low scores for non-hypermutated tumors. The magnitude of the differential expression between hypermutated and non-hypermutated tumors in the TCGA training datasets allowed a straightforward approach to this problem. Five genes with consistent up-regulation in hypermutated tumors and five with consistent down-regulation were selected. Each gene was given a weight equal to its mean t-statistic across the 3 datasets, and for each sample, the Hypermutation Predictor score was calculated as the sum of its weighted log2-transformed gene expression values. As the positive and negative weights were nearly balanced, the weights were rescaled such that they summed to 0, achieving a score that was invariant to any normalization scheme that adjusts each sample by a scaling constant (i.e., a sample’s score was the same under any housekeeping gene normalization regimen, or even in unnormalized data). As a final step, the score was centered and scaled by its mean and SD in MSS samples. As for the MMR Loss algorithm, care must be taken to translate these mean and SD scaling parameters across platforms.

To estimate the mean Hypermutation Predictor score in MSS tumors in a new dataset with unknown MSI status, two approaches can be used. In our validation datasets, we used model-based clustering under the assumption of a Gaussian mixture model to estimate this parameter without reference to known tumor MSI status. Alternatively, in datasets where MSI incidence is expected to be low, the median Hypermutation Predictor score will serve as a reasonable estimate of its mean in MSS tumors.

As with the MMR Loss algorithm, we made the assumption that the Hypermutation Predictor score’s SD in MSS tumors in TCGA would translate to future datasets. This assumption held imperfectly and resulted in a modest hit to the MSI Predictor algorithm’s performance, as detailed in the Discussion. The SD parameters derived in TCGA datasets are reported in Supplementary Table 2.

*Development of the MSI Predictor algorithm from the MMR Loss algorithm and the Hypermutation Predictor algorithm*

To develop a single algorithm that would integrate the MMR Loss and Hypermutation Predictor Algorithms, we exploited the fact that both the MMR Loss and Hypermutation Predictor scores were designed to be approximately Gaussian with a mean of 0 and SD of 1 in MSS samples. Furthermore, these two algorithms appeared uncorrelated in MSS samples. These observations suggested a test that rejects the null hypothesis of MSS/non-hypermutation in samples that fall in extreme values of the joint distribution of these two scores, which could be reasonably approximated as a bivariate normal distribution.

However, we desired a one-sided test and a rejection of the null hypothesis of MSS/non-hypermutation (e.g., when MLH1 expression was extremely high). Additionally, we wished to prevent a null score from one test from counteracting the evidence from an impressive score from the other test. (E.g., if the Hypermutation Predictor score suggested hypermutation but all the MMR genes were unusually high, we did not want to let the MMR genes’ results counteract the evidence from the Hypermutation Predictor score.) Thus both the MMR Loss score and the Hypermutation Predictor score were truncated at 0.

This truncation and the assumption of approximate bivariate normality lead to the following test statistic: MSI predictor score = [(max(HPS,0)^2^ + min(MLS,0)^2^)^1/2^], where HPS is the Hypermutation Predictor score and MLS is the MMR Loss score. Selected contours of this test score, or equivalently, decision boundaries it could delineate, are shown in Figure 3. Under the assumption of bivariate normality, a p-value was calculated for the test statistic that was equal to the mass of a bivariate normal probability distribution falling above the decision boundary implied by the test statistic’s value. Using numerical integration, p-values of 0.05, 0.01, 0.005, and 0.001 corresponded to test statistics of 2.058, 2.699, 2.939, and 3.429, respectively. The MMR Loss, Hypermutation Predictor, and MSI Predictor algorithms were all implemented in an R (R Foundation for Statistical Computing, Vienna, Austria) script. Formal descriptions of these algorithms follow below.

## Calibration of algorithms for diagnostic applications

The unsupervised procedure described in the Materials and Methods section for estimating mean expression in MMR-proficient tumors, although suitable for a retrospective analysis, is too unpredictable for a prospectively-defined diagnostic assay. For diagnostic applications, better performance could be achieved by directly estimating the mean and SD parameters in a large set of microsatellite stable (MSS) samples.

## Algorithm for calculation of the MMR Loss score

We propose the below algorithm for calling hypermutation events resulting from loss of expression of 1 of the 4 key MMR genes (MLH1, MSH2, MSH6, or PMS2).

1. Normalize the gene expression dataset using a sensible method, such as housekeeper normalization in low-to-medium-plex assays or global normalization in transcriptome-wide assays.
2. For each gene, estimate µ, the gene’s mean expression in non-hypermutated samples. If a low rate of hypermutation is expected in the dataset, each gene’s median expression provides a good estimate. If hypermutation is expected to be common, a Gaussian mixture model with two clusters can be fit to each gene’s expression data, and the mean of the higher expression cluster should be taken as µ. For single sample applications, µ must be pre-defined using a training dataset run on the same assay.
3. For each gene, look up its standard deviation (SD, [σ]) in non-hypermutated tumors of the appropriate cancer type in TCGA database. The 4 MMR genes’ σ values are provided in Supplementary Table 1.
4. For each sample, score each gene relative to its expected value in non-hypermutated samples as Z = (x-µ)/σ, where x is the gene’s normalized log2 expression value.
5. For each sample, call Zm the minimum Z score from the 4 genes. Calculate the final MMR Loss score, MLS = (Zm+1.03)/0.69, where 1.03 and 0.69 are the theoretical expectation and SD of the minimum of 4 standard normal random variables.
6. Calculate a p-value for each sample: p = Φ(MLS), where Φ is the standard normal distribution function. Choose a stringent p-value threshold for calling loss events, at least as strict as 0.01. Most loss of expression events are substantial enough that they are easily detected, so p-values between 0.05 and 0.01 will often result in false positives.

**Supplementary Table 1.** *Standard deviations of each mismatch repair gene in MSS samples in TCGA databases.*

| **Tumor Type** | **MLH1** | **MSH2** | **MSH6** | **PMS2** |
| --- | --- | --- | --- | --- |
| COAD | 0.3241 | 0.4108 | 0.4198 | 0.3259 |
| STAD | 0.4245 | 0.6020 | 0.4814 | 0.4314 |
| UCEC | 0.4543 | 0.7312 | 0.6158 | 0.4217 |

Note: COAD = colon adenocarcinoma; STAD = stomach adenocarcinoma; UCEC = uterine corpus endometrial carcinoma.

## Algorithm for calculation of the Hypermutation Predictor score.

We propose the below algorithm for calling hypermutation events from genes that are differentially expressed between hypermutated/ tumors with microsatellite instability (MSI) and non-hypermutated/MSS tumors.

1. For a given sample, Log2-transform the expression data for each of the genes in Table 2, multiply each gene by its given weight, and take the sum of these weighted expression values. Call this value x.
2. If one is applying the assay to a new platform, calibration of the mean parameter for that specific dataset is needed: for this purpose, fit a Gaussian mixture model with two classes to the data at hand, and take the lower of the two mean parameters. If one has previously estimated the mean parameter for that specific platform, that value can be used instead. Call the mean parameter µ.
3. Look up the score’s SD (σ) in non-hypermutated tumors of the appropriate cancer type in TCGA datasets. The 4 datasets’ σ values are provided in Supplementary Table 2.
4. Z-transform the score to have a mean of 0 and SD of 1 in non-hypermutated sample: calculate the Hypermutation Predictor score, HPS = (x-µ)/σ.
5. for each sample: p = Φ(HPS), where Φ is the standard normal distribution function. Choose a stringent p-value threshold for calling loss events, at least as strict as 0.01.

**Supplementary Table 2.** *Standard deviations of the Hypermutation Predictor score in MSS samples in TCGA datasets.*

| **Tumor Type** | **SD** |
| --- | --- |
| COAD | 0.6604 |
| STAD | 0.8153 |
| UCEC | 0.7027 |

Note: COAD = colon adenocarcinoma; SD = standard deviation; STAD = stomach adenocarcinoma; UCEC = uterine corpus endometrial carcinoma.

## Algorithm for calculation of the MSI Predictor score from the MMR Loss and Hypermutation Predictor scores

We propose the below algorithm for calling MSI-H status in a given sample:

1. Calculate the MMR Loss and Hypermutation Predictor scores as described above. Call MLS the Z-score from the MMR Loss algorithm, and call HPS the Z-score from the Hypermutation Predictor algorithm.
2. Calculate the final score: MSI Predictor Score = (max(HPS,0)^2 + min(MLS,0)2)^0.5.
3. Compare the score to a pre-specified cutoff. We suggest a cutoff of 2.058, which corresponds to a p = 0.01 threshold for rejecting the null hypothesis of MSS/non-hypermutation.
