# Supplementary figures and images for: Correction to: A gene expression assay for simultaneous measurement of microsatellite instability and anti-tumor immune activity
Source: J Immunother Cancer. 2019 Mar 15;7:76. doi: 10.1186/s40425-019-0560-x (PMC6419377; doi:10.1186/s40425-019-0560-x)

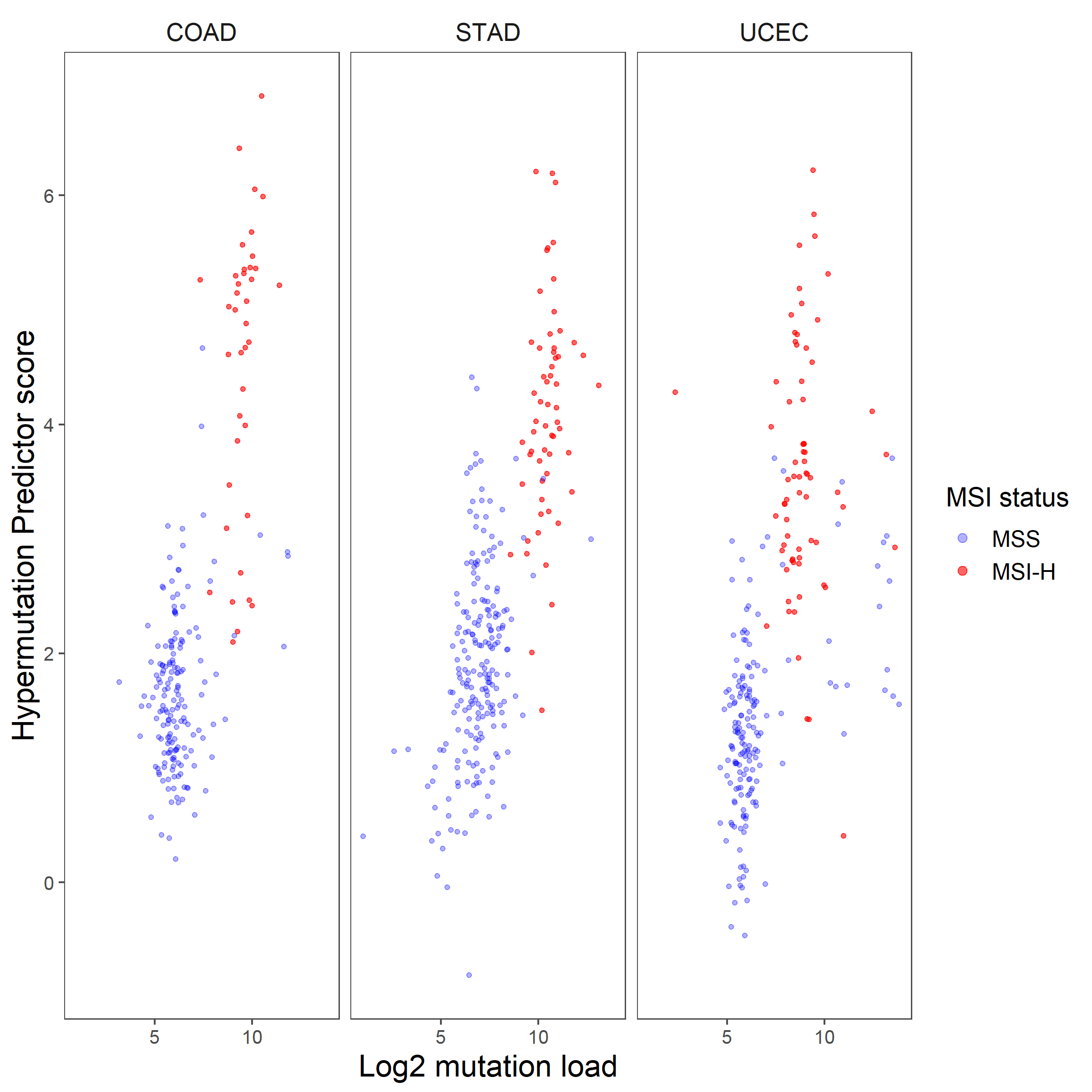

Supplement: Supplementary file 1 — Code and data for training analysis in TCGA data. The R code and data used in the TCGA analyses are included in this zip file. Code executes in the directory in which it is placed. (ZIP 120895 kb) [file 40425_2019_560_MOESM1_ESM.zip › tcga analysis/plots/Additional File 17 - TMB vs HPS.tiff]

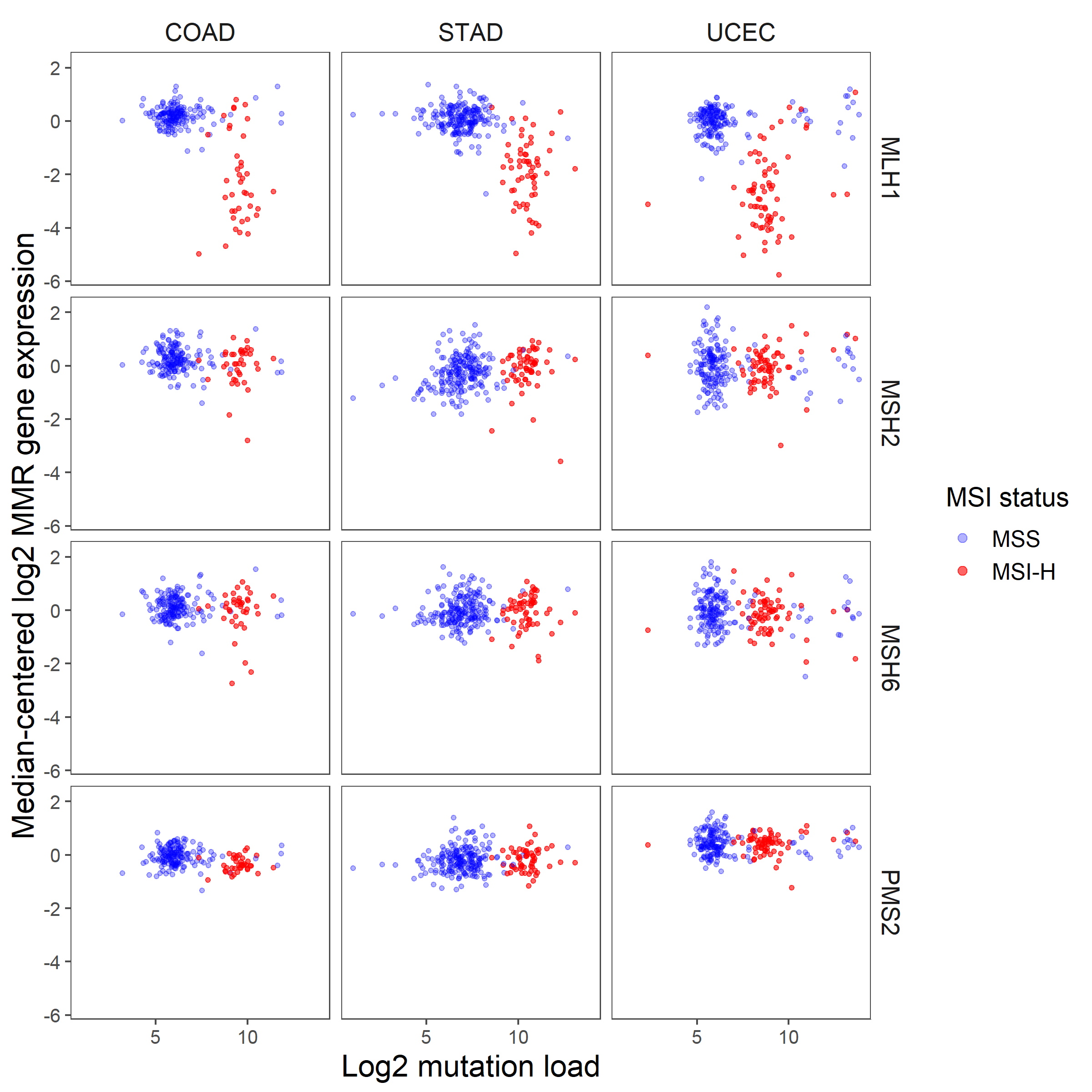

Supplement: Supplementary file 1 — Code and data for training analysis in TCGA data. The R code and data used in the TCGA analyses are included in this zip file. Code executes in the directory in which it is placed. (ZIP 120895 kb) [file 40425_2019_560_MOESM1_ESM.zip › tcga analysis/plots/Figure 1 - key4 vs mut.tiff]

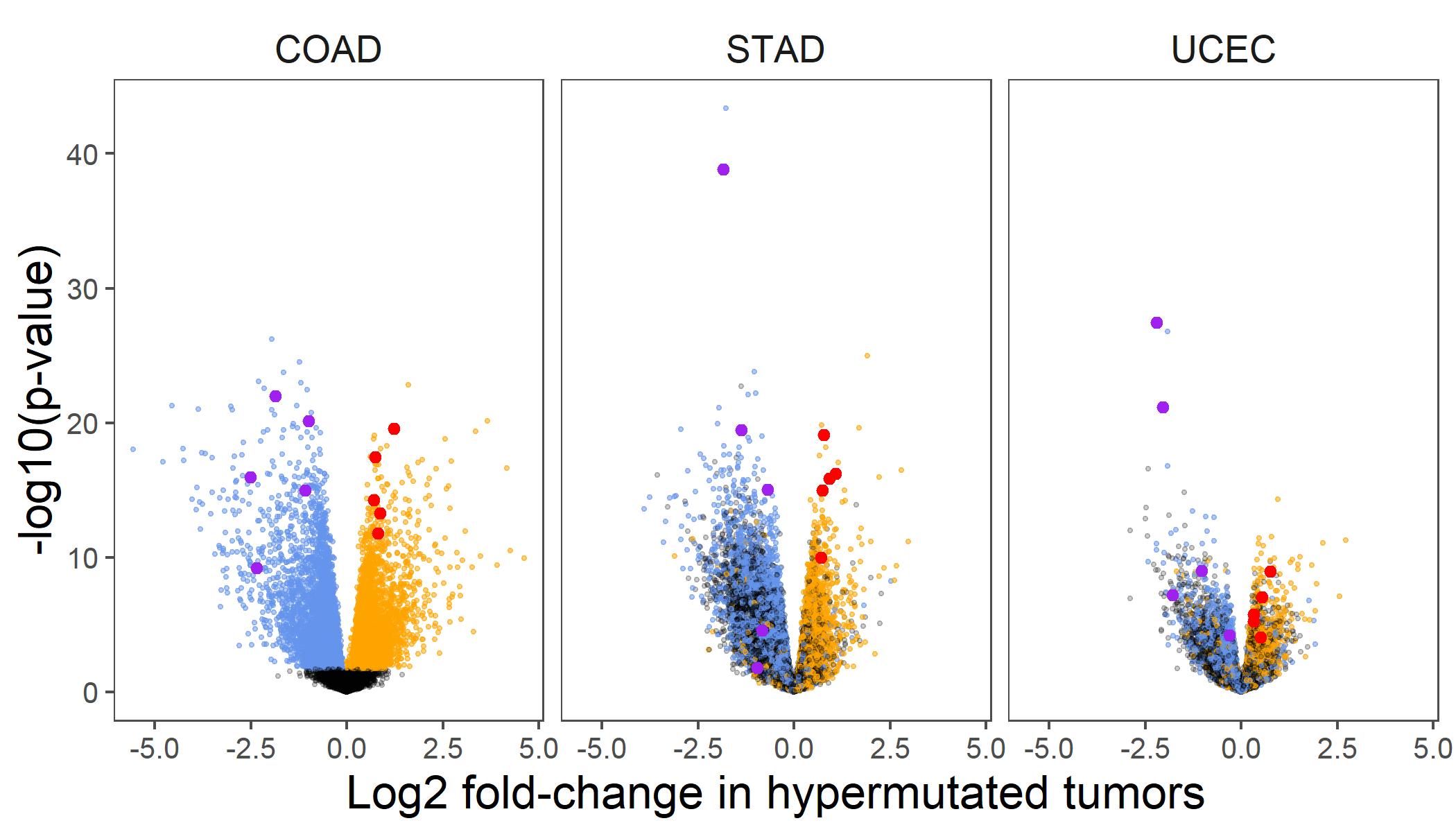

Supplement: Supplementary file 1 — Code and data for training analysis in TCGA data. The R code and data used in the TCGA analyses are included in this zip file. Code executes in the directory in which it is placed. (ZIP 120895 kb) [file 40425_2019_560_MOESM1_ESM.zip › tcga analysis/plots/Figure 2 - volcano plots.tiff]

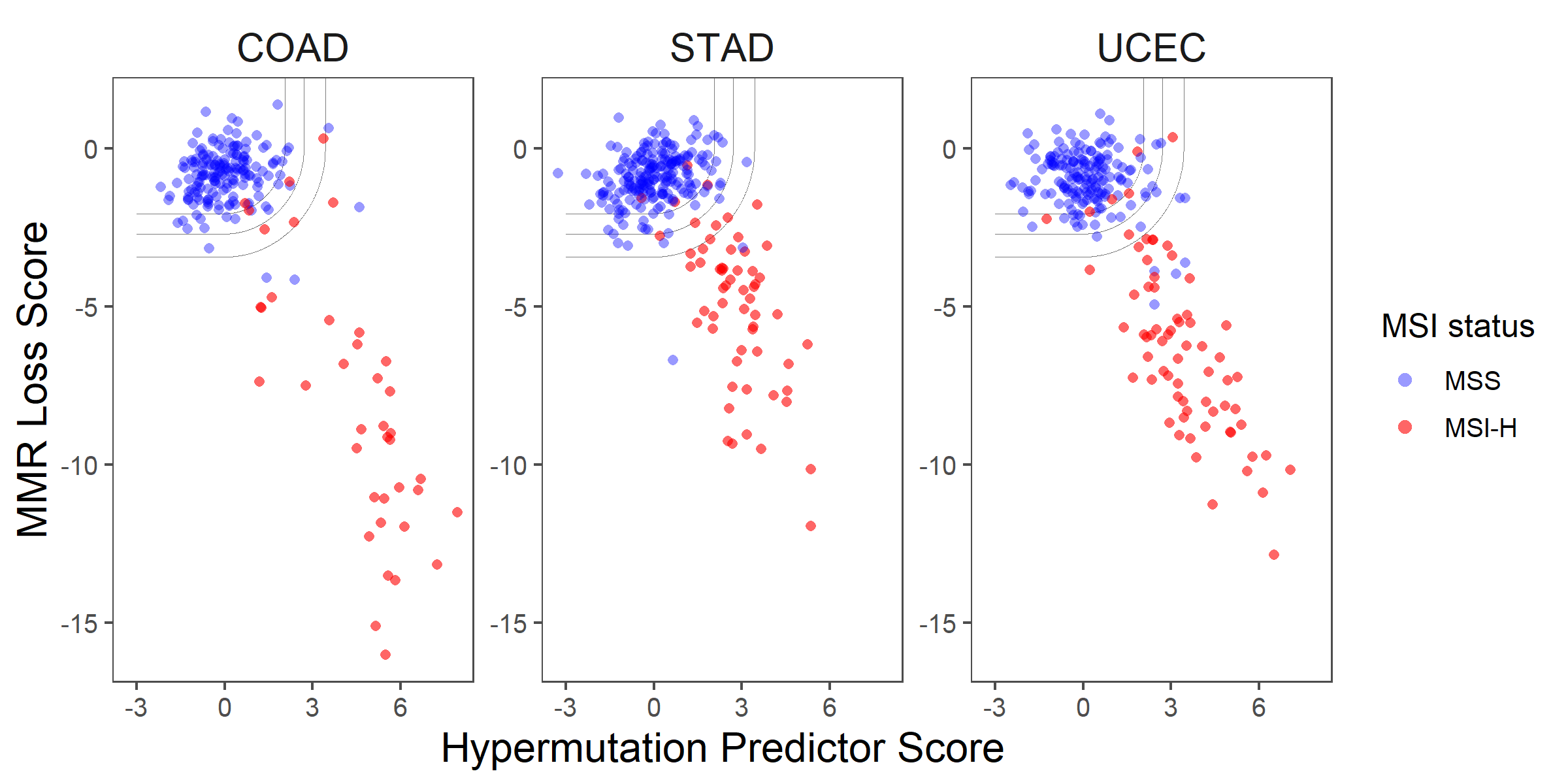

Supplement: Supplementary file 1 — Code and data for training analysis in TCGA data. The R code and data used in the TCGA analyses are included in this zip file. Code executes in the directory in which it is placed. (ZIP 120895 kb) [file 40425_2019_560_MOESM1_ESM.zip › tcga analysis/plots/Figure 3 - DE score vs LOE scores.tiff]

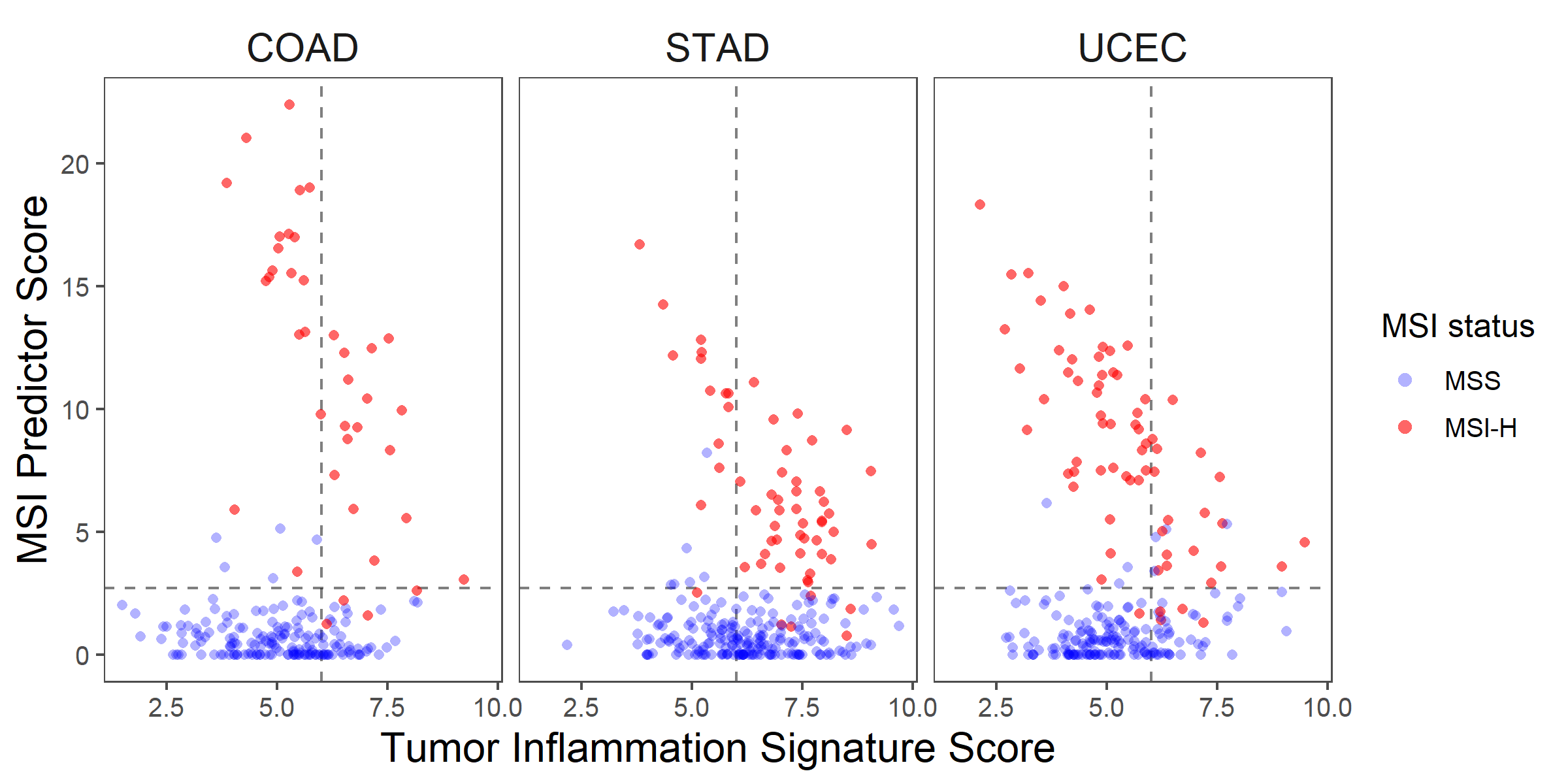

Supplement: Supplementary file 1 — Code and data for training analysis in TCGA data. The R code and data used in the TCGA analyses are included in this zip file. Code executes in the directory in which it is placed. (ZIP 120895 kb) [file 40425_2019_560_MOESM1_ESM.zip › tcga analysis/plots/Figure 5 - TIS vs. MSI score.tiff]

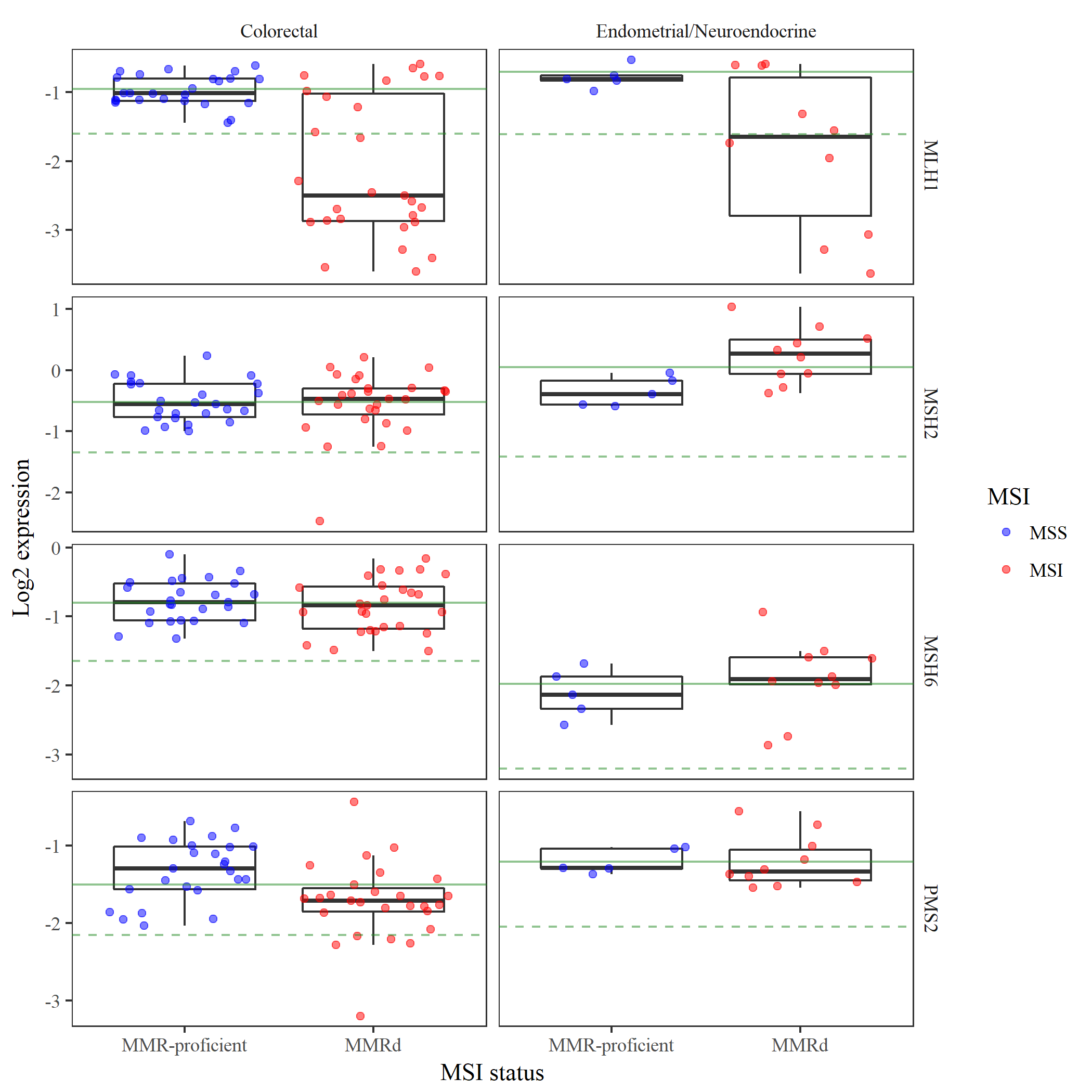

Supplement: Supplementary file 2 — Code and data for the validation dataset analyses. The R code and data used in the colorectal and endometrial/neuroendocrine validation analyses are included in this zip file. Code executes in the directory in which it is placed. (ZIP 456 kb) [file 40425_2019_560_MOESM2_ESM.zip › validation with CRC and endo data/supplemental figure 1 - MMR genes vs. MSI in validation.tiff]
